# Supplementary material for: High-fidelity topochemical polymerization in single crystals, polycrystals, and solution aggregates
Source: Nat Commun. 2025 Apr 12;16:3498. doi: 10.1038/s41467-025-58822-2 (PMC11993752; doi:10.1038/s41467-025-58822-2)
Supplement: Supplementary file 1 — Supplementary Information [file 41467_2025_58822_MOESM1_ESM.pdf]

Supplementary Information

High-Fidelity Topochemical Polymerization in Single Crystals, Polycrystals, and Solution Aggregates

Chongqing Yang[1#], Jianfang Liu[1#], Rebecca Shu Hui Khoo[1], Maged Abdelsamie[1+], Miao Qi[1], He Li[1,2], Haiyan Mao[3], Sydney Hemenway[1], Qiang Xu[4], Yunfei Wang[1,5,6], Beihang Yu[1], Qingsong Zhang[1], Xinxin Liu[7], Liana M Klivansky[1], Xiaodan Gu[6], Chenhui Zhu[5], Jeffrey A. Reimer[3], Ganglong Cui[7], Carolin M. Sutter-Fella[1], Jian Zhang[1], Gang Ren[1], Yi Liu[1,2*]

Affiliations:

1. The Molecular Foundry, Lawrence Berkeley National Laboratory, Berkeley, CA, 94720, USA;
2. Materials Sciences Division, Lawrence Berkeley National Laboratory, Berkeley, CA, 94720 USA;
3. Department of Chemical and Biomolecular Engineering, University of California, Berkeley, CA, 94720, USA;
4. Chemical Science Division, Lawrence Berkeley National Laboratory, Berkeley, CA, 94720, USA;
5. Advanced Light Source, Lawrence Berkeley National Laboratory, Berkeley, CA, 94720 USA;
6. School of Polymer Science and Engineering Center for Optoelectronic Materials and Devices, The University of Southern Mississippi, Hattiesburg, MS, 39406 USA;
7. Key Laboratory of Theoretical and Computational Photochemistry, Ministry of Education, Chemistry College, Beijing Normal University, Beijing, 100875 P.R. China;

[#] C. Yang and J. Liu contributed equally to this work;

[+] Current address: Interdisciplinary Research Center for Intelligent Manufacturing and Robotics, King Fahd University of Petroleum and Minerals (KFUPM), Dhahran, 31261 Saudi Arabia

[*] Corresponding author: Dr. Yi Liu; Email: yliu@lbl.gov

Table of Contents

1. Supplementary Methods 3

2. Supplementary Figures 11

3. Supplementary Tables.. 53

4. NMR spectra of CM1-s/r and CM2-s/r 57

5. Reference 64

1. Supplementary Methods

1.1 General Information

All the starting materials are purchased from Sigma Aldrich or TCI America. The monomer syntheses were carried out via Schleck-line techniques under nitrogen atmosphere unless otherwise stated. Column chromatography was performed using silica gel.

1.2 General Characterization

Solution ^{1}H and ^{13}C NMR spectra were recorded on a Bruker Avance II 500 spectrometer at frequencies of 500.12 MHz and 125.03 MHz by using the deuterated solvents (d_6-DMSO; $CDCl_3$ and CD_2Cl_2; the internal reference: tetramethylsilane). Solid-state ^{13}C cross-polarization magic angle spinning (CP-MAS) NMR spectra were acquired using an AVANCE NEO console operating at a magnetic field strength of 9.4 T (400.1 MHz for ^{1}H). A contact time of 5 ms was employed with high-power ^{1}H decoupling. MAS experiments utilized 3.2-mm MAS probes spinning at 20 kHz. The ^{13}C signals were referenced to the methylene signal of adamantane at 38.5 ppm. MALDI-TOF mass spectrometry tests were conducted on an Applied Biosystems 4800 MALDI TOF/TOF with super-DHB as the matrix. Fourier transform infrared spectroscopy (FTIR) was obtained on a Spectrum 100 (Perkin Elmer, Inc., USA) spectrometer with a scan range of 4000-400 cm^{-1}. Thermalgravimetric analysis (TGA) was performed on a Q5000IR thermosgravimetric analyzer under argon atmosphere with a heating rate of 10 °C/min. Differential Scanning Calorimetry (DSC) were recorded on a TA Q200 instrument with a heat rate of 10 °C min^{-1}. Both solid and solution Ultraviolet-Visible (UV-vis) spectra were recorded on a Varian 5000 Spectrophotometer. Powder X-ray diffraction (XRD) patterns were recorded on a Rigaku MiniFLex 6G Benchtop XRD using Cu-Kα radiation (λ = 0.15406 nm) at 40 kV. Optical microscope (OM) was performed on Zeiss with a Hitachi KP-D50 color digital CCD camera. Scanning electron microscopy (SEM) images were obtained from a Zeiss Gemini Ultra-55 Analytical Field Emission Scanning Electron Microscope via the in-lens detector. AFM images were acquired on a Bruker Dimension Icon AFM, with either a noncontact tapping mode or PeakForce tapping mode. Crystal on Si wafers were observed. Dynamic light scattering (DLS) measurements were conducted using a Malvern Analytical Zetasizer Nano-

ZS instrument. All samples were analyzed in a plastic cuvette at room temperature with a 633 nm laser source. The scattering angle was set at 173 °. The refractive index of the solute was 2.3403, and the refractive index of the solvent (water) was 1.33. Data analysis was performed using Zetasizer software version 7.13.

1.3 Monomer synthesis

1,4-Diacetylpiperazine-2,5-ione (DKP) was prepared according to previously reported method without modification. [1]

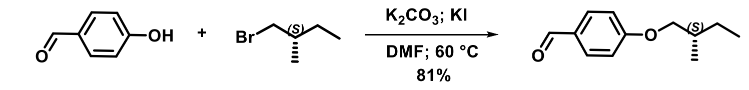

4-hydroxybenzaldehyde (S)-(+)-1-Bromo-2-methylbutane (S)-4-(2-methylbutoxy)benzaldehyde

Synthesis of (*S*)-4-(2-methylbutoxy)benzaldehyde.

To a 100 mL round-bottom flask under N_2 atmosphere was added 4-hydroxylbenzaldehyde (1.8 g, 14.7 mmol), potassium carbonate (6.1 g, 44.2 mmol) and potassium iodide (130 mg, 0.7 mmol), followed by the addition of a DMF solution (80 mL) of (*S*)-(+)-1-bromo-2-methylbutane (2.0 mL, 16.2 mmol). After heating at 60 ºC for overnight, the suspension was filtered, and the solvent was evaporated. The residue was further purified by column chromatography (hexanes/ethyl acetate = 3/1), affording (*S*)-4-(2-methylbutoxy)benzaldehyde in 80% yield. ^{1}H NMR (500 MHz, $CDCl_3$): δ 9.90 (s, 1H), 7.84-7.87 (m, 2H), 7.01-7.04 (m, 2H), 3.91-3.94 (m, 1H), 3.83-3.86 (m, 1H), 1.88-1.97 (m, 1H), 1.56-1.64 (m, 1H), 1.27-1.34 (m, 1H), 1.05 (d, J = 1.1 Hz, 3H), 0.97 (t, J = 1.0 Hz, 3H). ^{13}C NMR (126 MHz, $CDCl_3$): δ 190.88, 164.45, 132.01, 129.72, 114.78, 73.20, 34.62, 26.07, 16.48, 11.31.

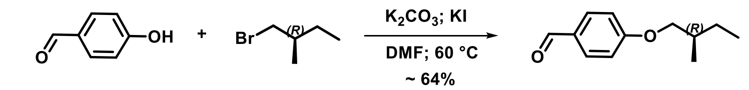

4-hydroxybenzaldehyde (R)-(-)-1-Bromo-2-methylbutane (R)-4-(2-methylbutoxy)benzaldehyde

Synthesis of (*R*)-4-(2-methylbutoxy)benzaldehyde.

The synthesis of (*R*)-4-(2-methylbutoxy)benzaldehyde followed the same procedure as above. The product was obtained in 64% yield. ^{1}H NMR (500 MHz, $CDCl_3$): δ 9.91 (s, 1H), 7.85-7.86 (m, 2H),

7.01-7.03 (m, 2H), 3.91-3.94 (m, 1H), 3.83-3.86 (m, 1H), 1.88-1.97 (m, 1H), 1.56-1.64 (m, 1H), 1.27-1.34 (m, 1H), 1.05 (d, *J* = 1.1 Hz, 3H), 0.97 (t, *J* = 1.0 Hz, 3H).

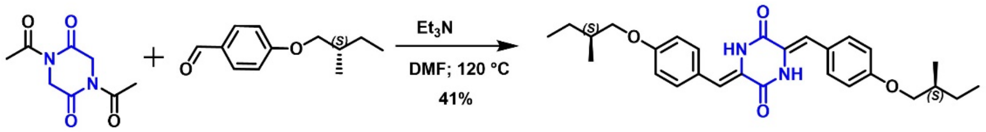

DKP (S)-4-(2-methylbutoxy)benzaldehyde CM1-*sa*

Synthesis of 3,6-bis((*Z*)-4-((*S*)-2-methylbutoxy)benzylidene)piperazine-2,5-dione (CM1-*sa*). In a 5 mL micro-wave vial purged with N_2, triethylamine (0.15 mL, 1.06 mmol, 3 eq) was added dropwise to a mixture of DKP (70 mg, 0.35 mmol, 1 eq), (*S*)-4-(2-methylbutoxy)benzaldehyde (150 mg, 0.78 mmol, 2.2 eq) and anhydrous DMF (1 mL). The vial was sealed, stirred and heated at 120 °C for 12 h. After cooling to room temperature, 5 mL water was added to the mixture. The resulting precipitate was filtered and recrystallized from methanol, affording the product as pale yellow powder in 41% yield. ^{1}H NMR (500 MHz, CD_2Cl_2): δ 8.19 (s, 2H), 7.40-7.43 (m, 4H), 7.01-7.04 (m, 4H), 6.94 (s, 2H), 3.89-3.92 (m, 2H), 3.80-3.83 (m, 2H), 1.87-1.96 (m, 2H), 1.57-1.65 (m, 2H), 1.28-1.36 (m, 2H), 1.05 (d, *J* = 1.1 Hz, 6H), 0.98 (t, *J* = 1.0 Hz, 6H). ^{13}C NMR (126 MHz, CD_2Cl_2): δ 159.75, 157.08, 130.01, 125.01, 124.58, 115.68, 115.39, 73.02, 34.68, 26.06, 16.20, 11.08.

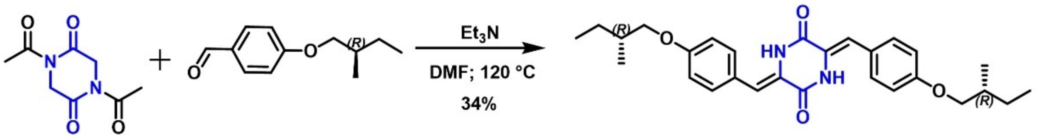

DKP (R)-4-(2-methylbutoxy)benzaldehyde CM1-*ra*

Synthesis of 3,6-bis((*Z*)-4-((*R*)-2-methylbutoxy)benzylidene)piperazine-2,5-dione (CM1-*ra*). The synthesis of CM1-*r*a was identical to that of CM1-*s*a. The product was obtained in 34% yield. ^{1}H NMR (500 MHz, $CDCl_3$): δ 8.14 (s, 2H), 7.36-7.38 (m, 4H), 6.99-7.01 (m, 6H), 3.87-3.90 (m, 2H), 3.80-3.83 (m, 2H), 1.87-1.96 (m, 2H), 1.57-1.65 (m, 2H), 1.27-1.35 (m, 2H), 1.05 (d, *J* = 1.1

Hz, 6H), 0.99 (t, *J* = 1.0 Hz, 6H).

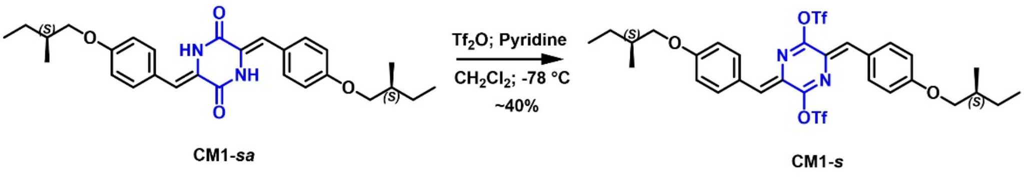

Synthesis of 3,6-bis((*Z*)-4-((*S*)-2-methylbutoxy)benzylidene)-3,6-dihydropyrazine-2,5-diyl bis(trifluoromethanesulfonate) (CM1-*s*).

A suspension of **CM1-*sa*** (75 mg, 0.16 mmol), pyridine (0.1 mL) and dichloromethane (6.5 mL) was cooled to -78 °C (dry ice/acetone bath), followed by the dropwise addition of trifluoromethanesulfonic anhydride (0.1 mL). The reaction mixture was stirred at -78 ºC for around 1h, and then was allowed to slowly warm to room temperature. After stirring for an additional 15 h, hexanes was added to the reaction mixture. The precipitate was collected, and washed with water and methanol to give CM1-*s* as orangish solid (yield: 40%). ^{1}H NMR (500 MHz, CD_2Cl_2): δ 8.06 (d, *J* = 8.0 Hz, 4H), 6.99 (d, *J* = 7.0 Hz, 4H), 6.91 (s, 2H), 3.93-3.96 (m, 2H), 3.84-3.87 (m, 2H), 1.87-1.97 (m, 2H), 1.57-1.66 (m, 2H), 1.28-1.37 (m, 2H), 1.06 (d, J = 1.1 Hz, 6H), 0.98 (t, J = 1.0 Hz, 6H). ^{13}C NMR (126 MHz, CD_2Cl_2): 162.04, 151.02, 134.31, 129.95, 124.32, 122.21, 119.66, 117.11, 114.88, 73.09, 34.66, 26.03, 16.18, 11.06.

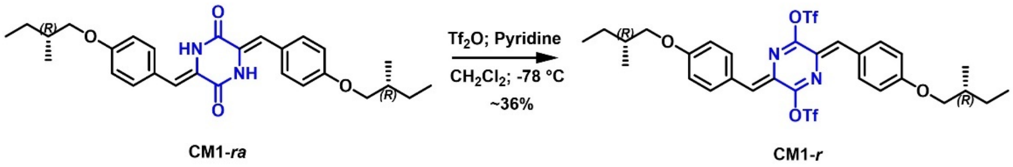

Synthesis of 3,6-bis((*Z*)-4-((*R*)-2-methylbutoxy)benzylidene)-3,6-dihydropyrazine-2,5-diylzbis(trifluoromethanesulfonate) (CM1-*r*).

CM1-*r* was synthesized using the same method as that for CM1-*s* and was obtained in 36% yield. ^{1}H NMR (500 MHz, $CDCl_3$): δ 8.01 (d, *J* = 8.0 Hz, 4H), 6.96 (d, *J* = 7.0 Hz, 4H), 6.86 (s, 2H), 3.90-

3.93 (m, 2H), 3.82-3.85 (m, 2H), 1.88-1.95 (m, 2H), 1.52-1.64 (m, 2H), 1.26-1.35 (m, 2H), 1.05 (d, J = 1.1 Hz, 6H), 0.99 (t, J = 1.0 Hz, 6H). ^{13}C NMR (126 MHz, $CDCl_3$): 161.98, 150.97, 134.42, 129.58, 126.02, 124.37, 121.14, 119.68, 117.12, 114.96, 73.05, 34.66, 26.09, 16.50, 11.32.

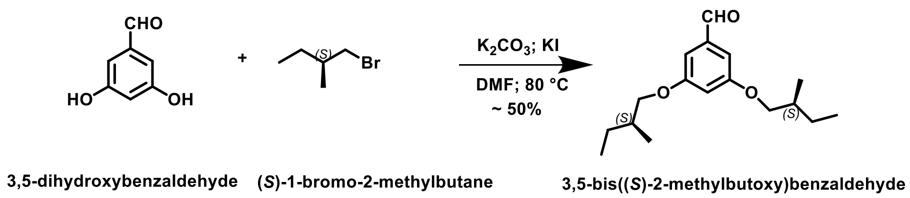

Synthesis of 3,5-bis((*S*)-2-methylbutoxy)benzaldehyde.

To a 50 mL round-bottom flask was added (*S*)-1-bromo-2-methylbutane (1.84 g, 12.16 mmol, 2.4 eq), 3,5-dihydroxybenzaldehyde (700 mg, 5.07 mmol, 1.0 eq), potassium carbonate (1.75 g, 12.67 mmol 2.5 eq), potassium iodide (84.13 mg, 0.51 mmol, 0.1 eq) and DMF 9 (mL) under N_2. The reaction mixture was stirred at 80 °C for 15 h, followed by filtration and drying to give a crude residue. Further purification via silica column chromatography (hexanes:ethyl acetate 3:1) yielded 3,5-bis((*S*)-2-methylbutoxy)benzaldehyde in 50% yield. ^{1}H NMR (500 MHz, $CDCl_3$): δ 9.92 (s, 1H), 7.01 (d, J = 7.0 Hz, 2H), 6.73 (t, J = 6.7 Hz, 1H), 3.86-3.89 (m, 2H), 3.78-3.81 (m, 2H), 1.85-1.94 (m, 2H), 1.55-1.63 (m, 2H), 1.25-1.34 (m, 2H), 1.04 (d, J = 1.0 Hz, 6H), 0.98 (t, J = 1.0 Hz, 6H).

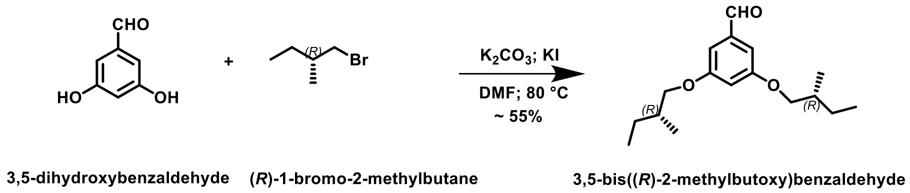

Synthesis of 3,5-bis((*R*)-2-methylbutoxy)benzaldehyde.

The synthesis was the same as that of 3,5-bis((*S*)-2-methylbutoxy)benzaldehyde. The product was obtained in 55% yield. ^{1}H NMR (500 MHz, $CDCl_3$): δ 9.92 (s, 1H), 7.01 (d, J = 7.0 Hz, 2H), 6.73 (t, J = 6.7 Hz, 1H), 3.86-3.89 (m, 2H), 3.78-3.81 (m, 2H), 1.85-1.93 (m, 2H), 1.55-1.63 (m, 2H), 1.23-1.36 (m, 2H), 1.04 (d, J = 1.0 Hz, 6H), 0.98 (t, J = 1.0 Hz, 6H).

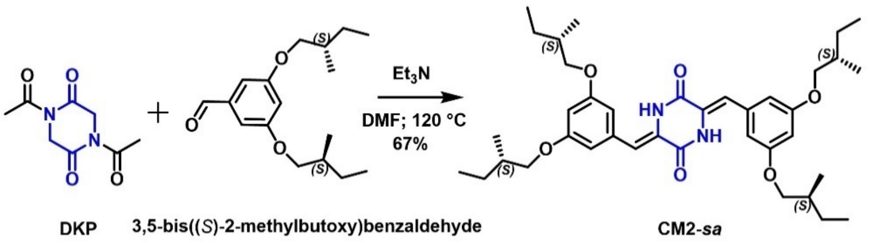

Synthesis of 3,6-bis((*Z*)-3,5-bis((*S*)-2-methylbutoxy)benzylidene)piperazine-2,5-dione (CM2-*sa*).

To a 3-neck round-bottom flask was added DKP (80 mg, 0.40 mmol, 1 eq), 3,5-bis((S)-2-methylbutoxy)benzaldehyde (236 mg, 0.85 mmol; 2.1 eq) and dry DMF (1 mL) under N_2 atmosphere. The mixture was stirred at 120 °C for 15 h. After cooling to room temperature, 10 mL methanol was added. The precipitate was filtered and washed with another 10 mL methanol, yielding the product as pale yellow powder (yield: 67%). ^{1}H NMR (500 MHz, $CDCl_3$): δ 8.24 (s, 1H), 6.97 (s, 2H), 6.48-6.49 (m, 6H), 3.82-3.85 (m, 4H), 3.74-3.77 (m, 4H), 1.86-1.93 (m, 4H), 1.56-1.64 (m, 4H), 1.25-1.34 (m, 4H), 1.04 (d, J = 1.0 Hz, 12H), 0.98 (t, J = 1.0 Hz, 12H).

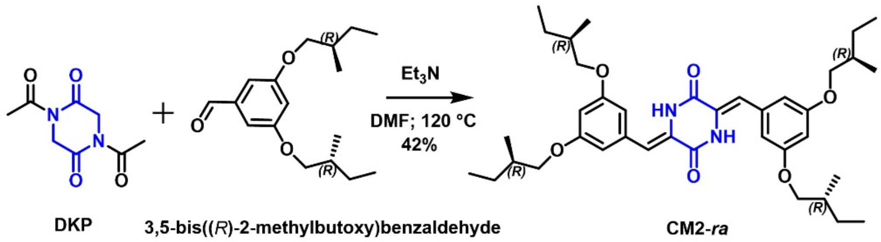

Synthesis of 3,6-bis((*Z*)-3,5-bis((*R*)-2-methylbutoxy)benzylidene)piperazine-2,5-dione (CM2-*ra*).

The synthesis of CM2-*ra* is identical to that of CM2-*sa*. The product was obtained in 42% yield. ^{1}H NMR (500 MHz, $CDCl_3$): δ 8.23 (s, 1H), 6.97 (s, 2H), 6.48-6.49 (m, 6H), 3.82-3.85 (m, 4H), 3.74-3.77 (m, 4H), 1.86-1.93 (m, 4H), 1.56-1.64 (m, 4H), 1.25-1.34 (m, 4H), 1.04 (d, J = 1.0 Hz, 12H), 0.98 (t, J = 1.0 Hz, 12H).

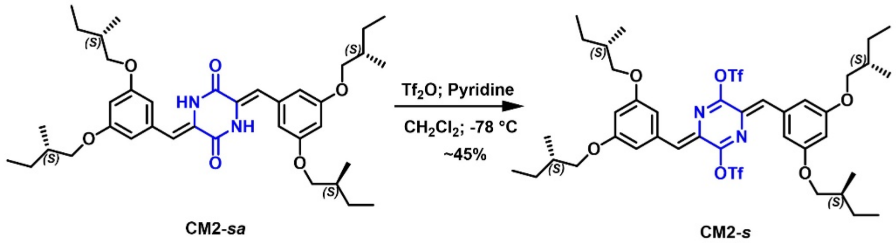

Synthesis of 3,6-bis((*Z*)-3,5-bis((*S*)-2-methylbutoxy)benzylidene)-3,6-dihydropyrazine-2,5-diyl bis(trifluoromethanesulfonate) (CM2-*s*).

A 3-neck round bottom flask was purged with N_2, followed by the addition of CM2-*s*a (70 mg) and dry DCM (4 mL). The reaction mixture was then cooled in the dry ice/acetone bath (-78 °C) for 15 min. Subsequently, pyridine (0.15 mL) and trifluoromethanesulfonic anhydride (0.2 mL) were added dropwise into the system. The reaction mixture was stirred for 12 h while being slowly warmed to above 0 °C. The reaction flask was kept at 5 °C in fridge for another 24 h, followed by the addition of 10 mL methanol to quench the reaction. The yellowish precipitate was filtered and washed with water and methanol for 3 times each, yielding CM2-*s* in 45% yield. ^{1}H NMR (500 MHz, CD_2Cl_2): δ 7.20 (d, J = 7.2 Hz, 4H), 6.91 (s, 2H), 6.62 (t, J = 6.6 Hz, 2H), 3.88-3.91 (m, 4H), 3.80-3.83 (m, 4H), 1.86-1.92 (m, 4H), 1.49-1.65 (m, 4H), 1.27-1.35 (m, 4H), 1.05 (d, J = 1.1 Hz, 12H), 0.98 (t, J = 1.0 Hz, 12H).

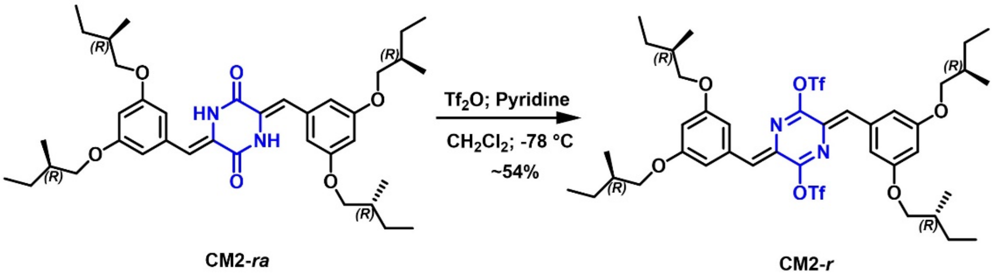

Synthesis of 3,6-bis((Z)-3,5-bis((S)-2-methylbutoxy)benzylidene)-3,6-dihydropyrazine-2,5-diyl bis(trifluoromethanesulfonate) (CM2-*r*).

CM2-*r* was synthesized using the same method as for CM2-*s* in 54% yield. ^{1}H NMR (500 MHz, $CDCl_3$): δ 7.17 (d, J = 7.2 Hz, 4H), 6.86 (s, 2H), 6.61 (t, J = 6.6 Hz, 2H), 3.86-3.89 (m, 4H), 3.79-3.82 (m, 4H), 1.85-1.92 (m, 4H), 1.57-1.65 (m, 4H), 1.25-1.34 (m, 4H), 1.00 (d, J = 1.1 Hz, 12H), 0.97 (t, J = 1.0 Hz, 12H). ^{13}C NMR (126 MHz, $CDCl_3$): 169.56, 151.14, 134.40, 131.67, 125.68,

121.78, 119.47, 116.93, 110.63, 105.84, 73.23, 34.82, 26.08, 16.46, 11.32.

Computational details

All geometry optimizations were performed using the B3LYP-D3 method.[2-6] The 6-31G* basis sets for all atoms.[2-8] Harmonic frequency analysis was carried out at the same level to verify the nature of minima. All DFT calculations were carried out using GAUSSIAN16.[9]

Crystallographic data and structural representation

Single crystal X-ray diffraction data were collected using either Bruker Venture *sc*-XRD or the synchrotron radiation at the Advanced Light Source, Berkeley CA. Indexing was performed using APEX3 (Difference Vectors method). Data integration and reduction were performed using SaintPlus 6.0. Absorption correction was performed by multi-scan method implemented in SADABS. Space groups were determined using XPREP implemented in APEX3.[10] The structure was solved using SHELXS-97 (direct methods) and refined using SHELXL-97 within Olex 2 (full-matrix least-squares on F^2). C, O, S, F, N atoms were refined with anisotropic displacement parameters and H atoms were placed in geometrically calculated positions and included in the refinement process using riding model with isotropic thermal parameters: $U_{iso}(H) = 1.2U_{eq}(-CH)$. The disordered solvent molecules were treated as diffuse using the SQUEEZE procedure implemented in PLATON. Crystal data and refinement details are shown in Table S1, S2, S3 and this data can be obtained free of charge from The Cambridge Crystallographic Data Centre via www.ccdc.cam.ac.uk/data_request/cif

2. Supplementary Figures

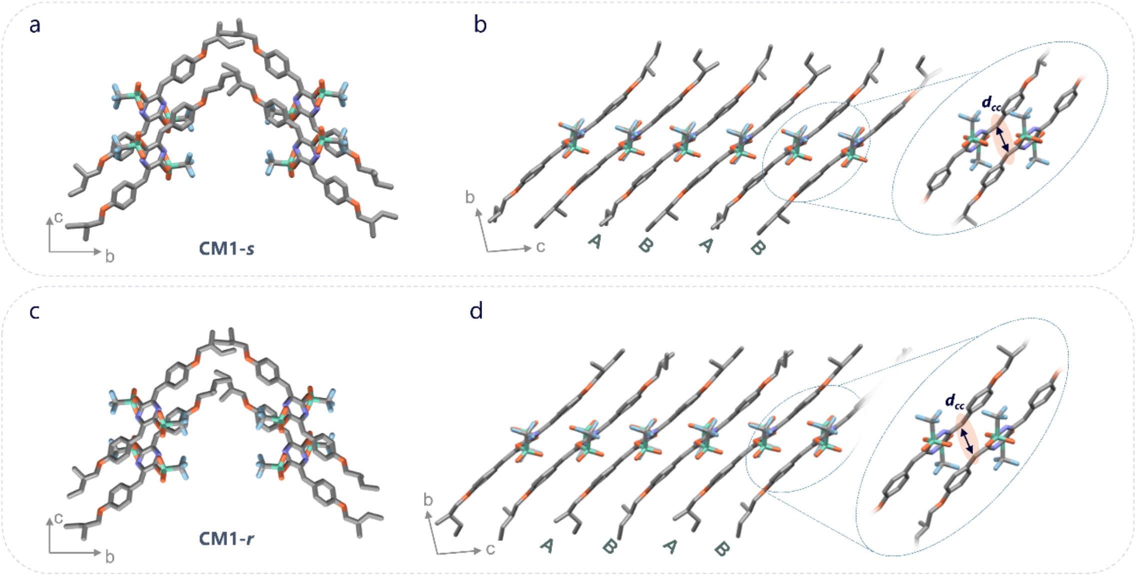

Supplementary Fig. S1| X-ray single crystal structures of chiral enantiomer CM1-*s* and CM1-*r*. a-b, Crystal packing of CM1-*s* viewed from different directions. **c-d,** Crystal packing of enantiomer CM1-*r* viewed from different directions. Note that in both CM1-*s* and CM1-*r*, the alkyl end groups from adjacent molecules within each column are oriented differently, leading to the ABAB columnar stacking and two distinct d_{cc} of 3.67 and 3.64 Å.

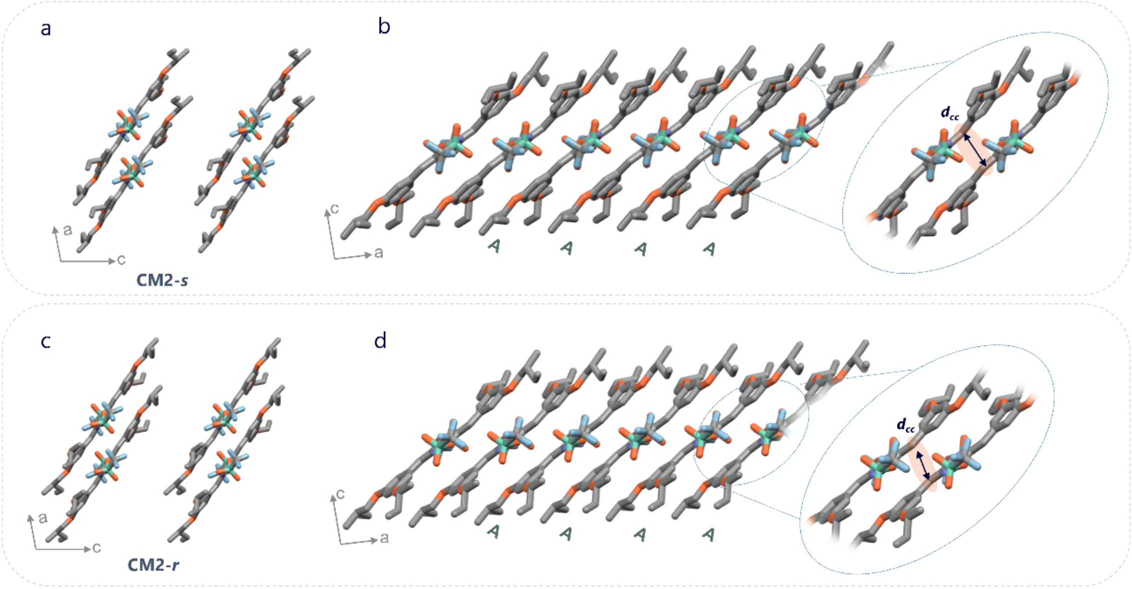

Supplementary Fig. S2| Single crystal X-ray structures of chiral enantiomer CM2-*s* and CM2-*r*. a-b, Crystal packing of CM2-*s* viewed from different directions. **c-d,** Crystal packing of enantiomer CM2-*r* viewed from different directions. All chiral side chains between adjacent monomers within each column are identical to each other, giving rise to one uniform d_{cc} of 3.65 Å.

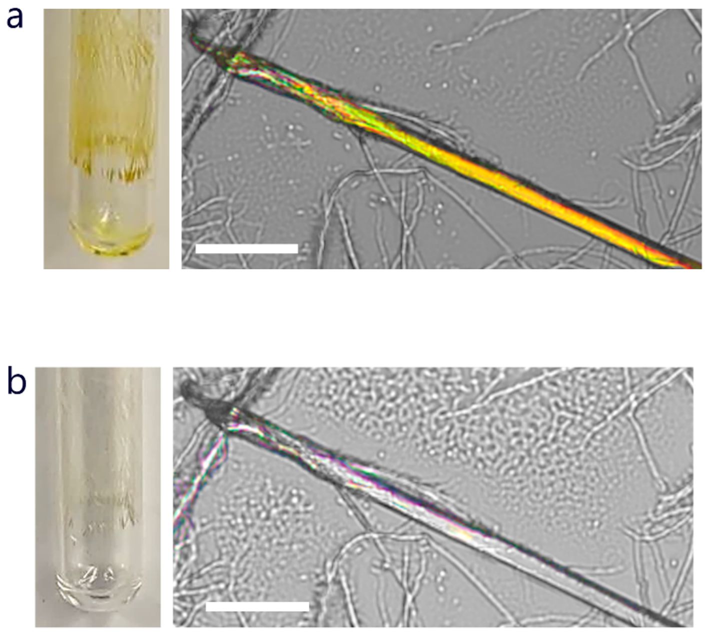

Supplementary Fig. S3| Photograph and optical microscopy of crystalline CM2-*s* before (a) and after (b) topochemical polymerization. Scale bar: 40 μm. The yellowish needle-like monomer crystals turned into colorless ones after ambient light irradiation for ~2 h at r.t..

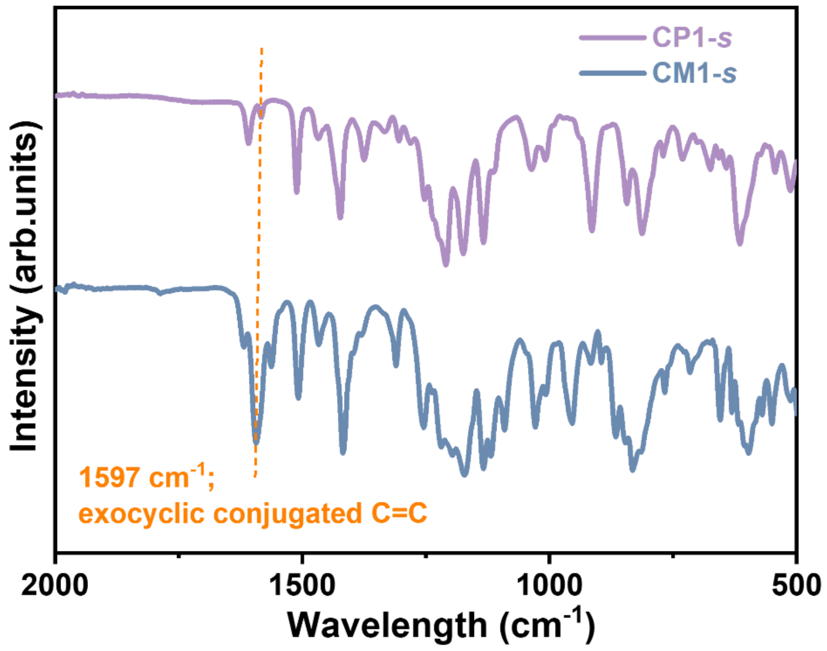

Supplementary Fig. S4| FTIR spectroscopic studies of CM1-*s* and CP1-*s*. The characteristic vibrational peak at 1597 cm^{-1} (C=C) for CM1-*s* disappeared in that for CP1-*s*, indicating the successful polymerization.

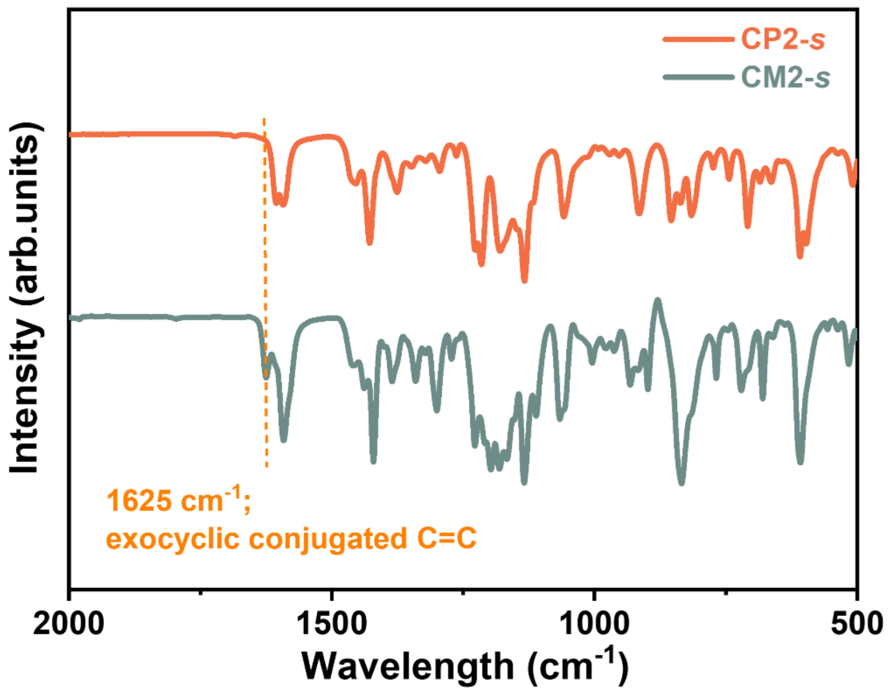

Supplementary Fig. S5| FTIR spectroscopic studies of CM2-*s* and CP2-*s*. Compared with that of CM2-*s*, the characteristic vibrational peak of C=C disappeared in that for CP2-*s*, indicating the successful polymerization.

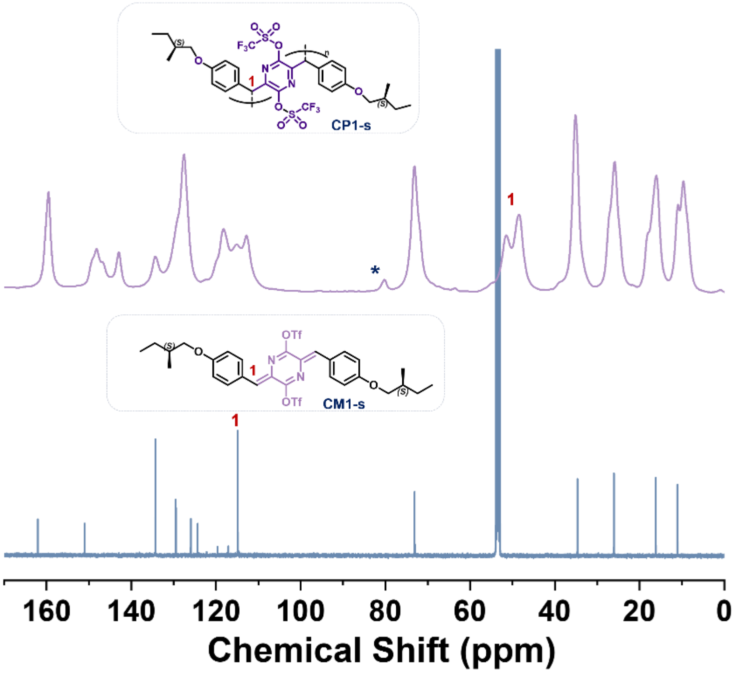

Supplementary Fig. S6| ^{13}C-NMR spectra of CM1-*s* (in CD_2Cl_2) and cross-polarization/magic angle spinning solid-state ^{13}C-NMR spectra of CP1-*s*. Compared with that of CM1-*s*, the appearance of peak at ~ 48 ppm (corresponding to xylyl carbon) indicated the formation of non-conjugated poly-*p*-xylylene derivative CP1-*s*.

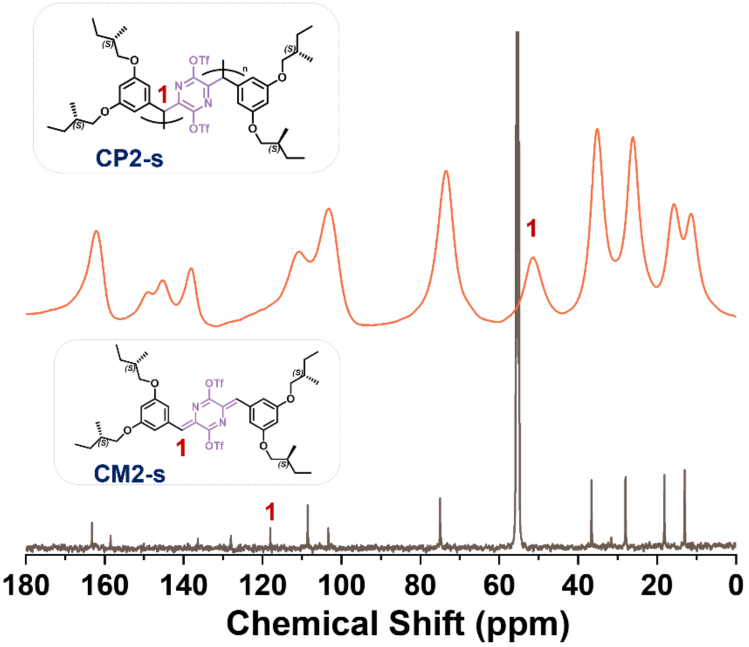

Supplementary Fig. S7| ^{13}C-NMR spectra of CM2-*s* (in CD_2Cl_2) and cross-polarization/magic angle spinning solid-state ^{13}C-NMR spectra of CP2-*s*. Compared with that of CM2-s, the appearance of peak at ~ 51 ppm (corresponding to xylyl carbon) indicated the formation of non-conjugated poly-*p*-xylylene derivative CP2-*s*.

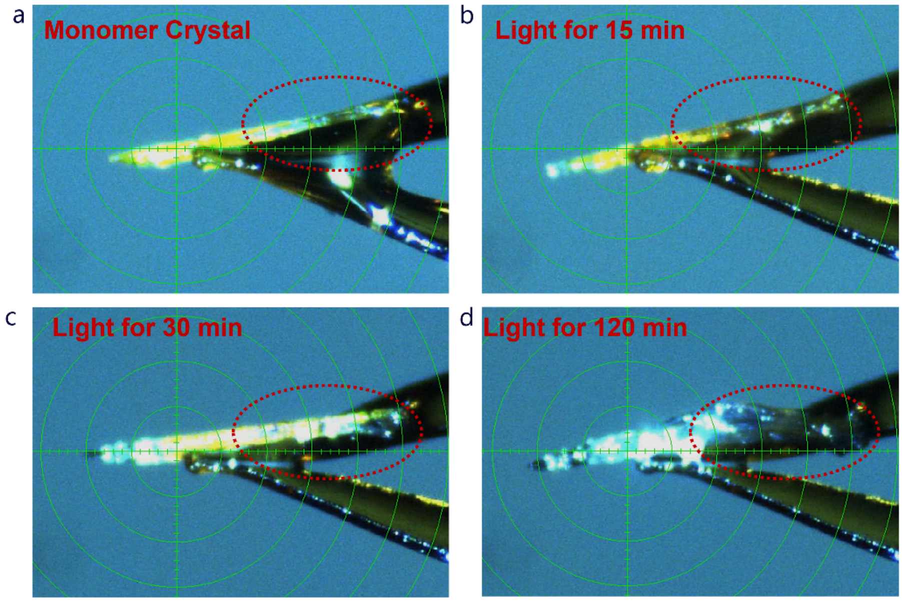

Supplementary Fig. S8| Optical microscopic images showing the morphology changes of a CM1-*s* single crystal during polymerization under ambient light. a, before exposure to light irradiation. **b**, after exposure for 15 min. **c**, after exposure for 30 min. **d**, after exposure for 120 min. The temperature was continuously changed from 100 K to 200 K**.** The crystal expanded and cracked into pieces, owing to the strains generated during the topochemical polymerization.

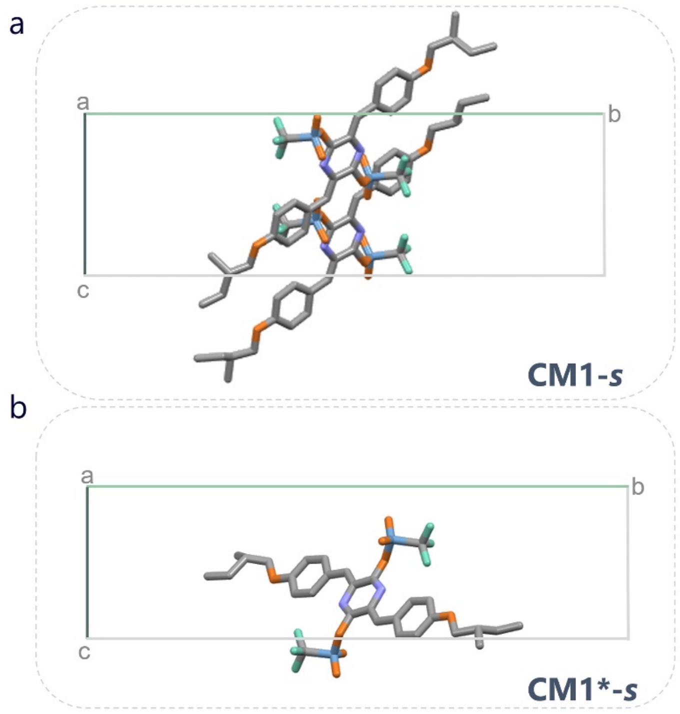

Supplementary Fig. S9| Single crystal structures of (a) CM1-*s* and (b) CM1*-*s* in each unit cell. The crystal of CM1-*s* contains two monomers per unit cell, while there is only one molecule in each unit cell for CM1*-*s*. H atoms are omitted for clarity.

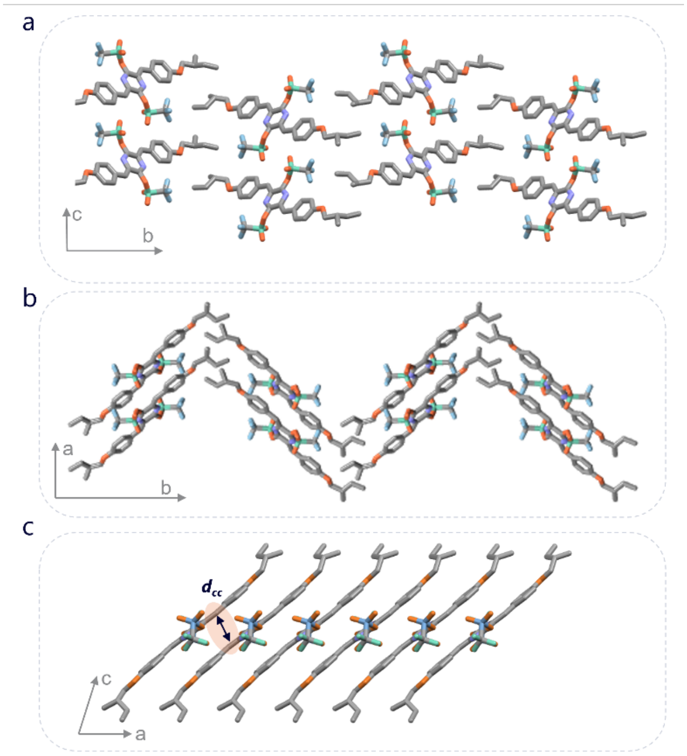

Supplementary Fig. S10| Single crystal structures of CM1*-*s* viewed from (a) a-axis, (b) c-axis, and (c) b-axis. H atoms are omitted for clarification. Compared with CM1-*s*, the lattice parameters of CM1*-*s* undergo significant alterations, with -14.2%, -0.1%, -44.7%, and -9.7% changes in a, b, c and β, respectively. Notably, the CM1*-*s* crystal has nearly half the cell volume as CM1-*s* (3212.9 vs 1608.6 Å^3).

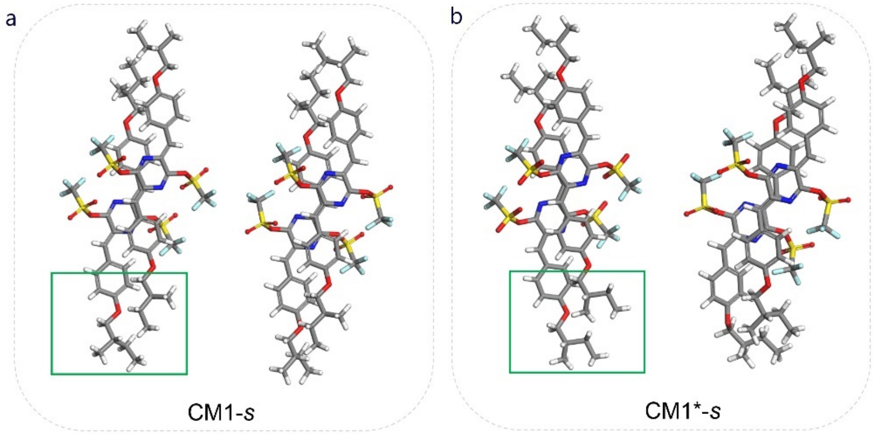

Relative Total Energy (kcal/mol) **0.0** **1.4**

Supplementary Fig. S11| The comparison of relative total energy for (a) CM1-*s* and (b) CM1*-*s* based on DFT calculations. CM1*-*s* has a higher energy of 1.4 kcal mol^{-1} than CM1-*s*.

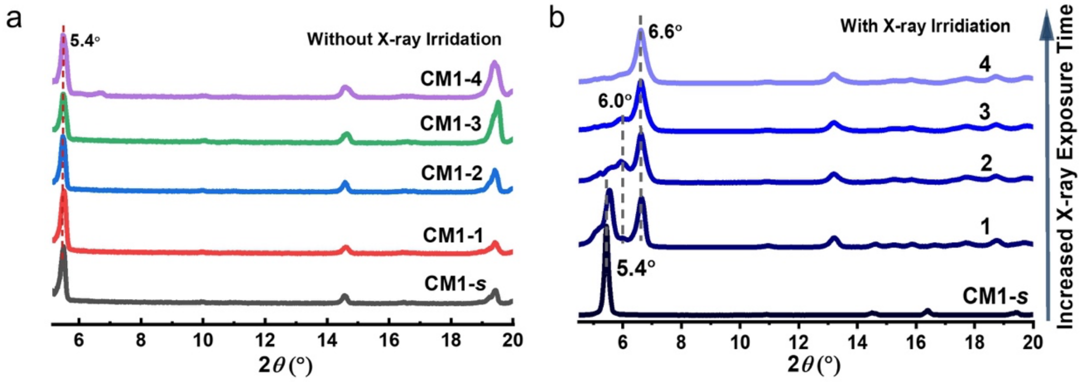

Supplementary Fig. S12| Time-dependent powder XRD spectra of CM1-s. a, CM1-*s* stored in the dark at r.t. without X-ray exposure, measured at time intervals of 0, 2, 4, 6 and 8 h, denoted as CM1-*s*, CM1-1, CM1-2, CM1-3 and CM1-4, respectively. **b**, Samples under continuous X-ray exposure. The number 1, 2, 3, 4 indicate cumulative X-ray irradiation durations of 2, 4, 6, and 8 hours, respectively.

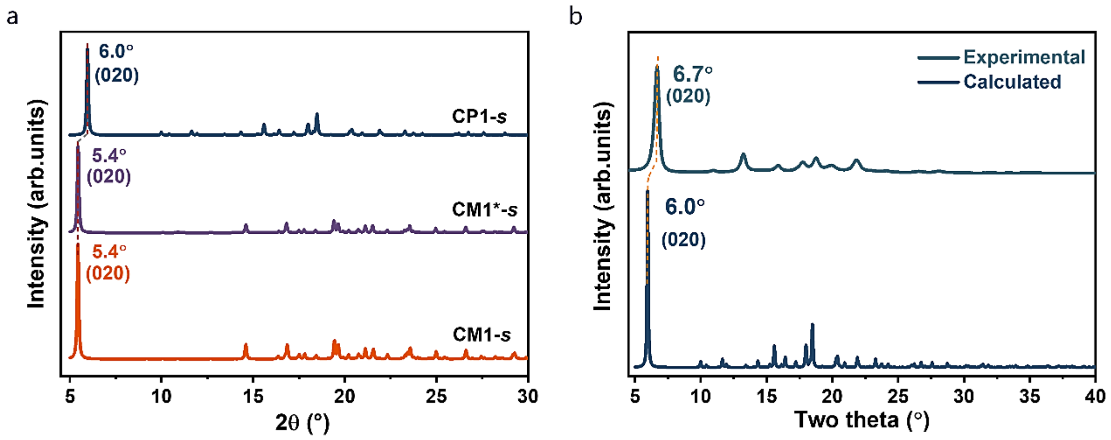

Supplementary Fig. S13| Theoretical and experimental PXRD of CM1-*s*, CM1*-*s* and CP1-*s*. **a,** Theoretical PXRD patterns of CM1-*s*, CM1*-*s* and CP1-*s* based on the single crystal structure from SCXRD analysis. **b,** Theoretical and experimental PXRD patterns of CP1-*s*. It should be noted that the (020) peak shift between experimental and theoretical one is caused by the temperature effect, since the experimental one was measured at 25 °C while the theoretical trace was based on SCXRD data acquired at 100 K.

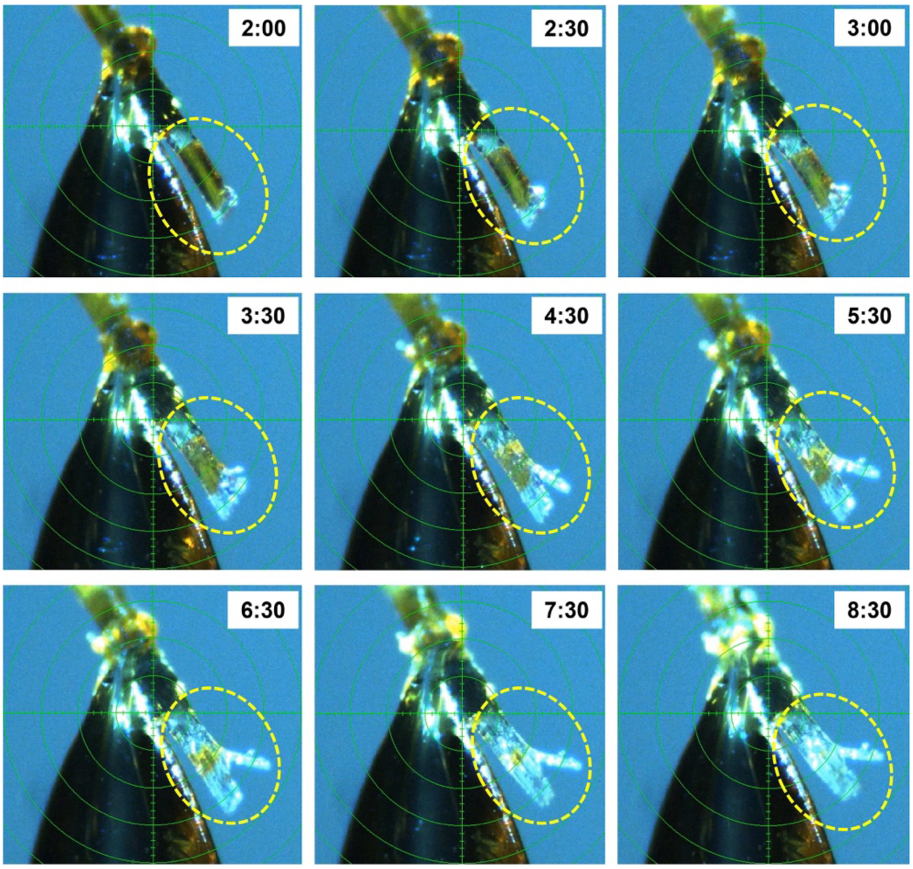

Supplementary Fig. S14|. Snapshots of the CM1-s crystal during the photo-induced polymerization. Under continuous exposure to ambient light at different time intervals (the unit is in minutes), it shows that the heterogeneous polymerization that led to crystal disintegration.

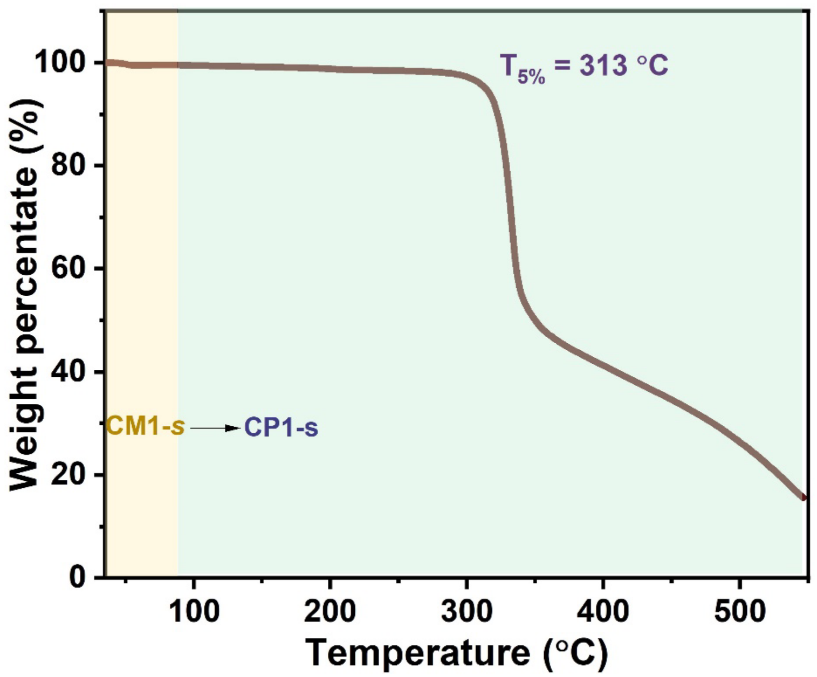

Supplementary Fig. S15| TGA characterization of CM1-*s* under nitrogen atmosphere. There is no noticeable weight loss before 313 °C ($T_{5\%}$), confirming the high thermal stability of CP1-*s*.

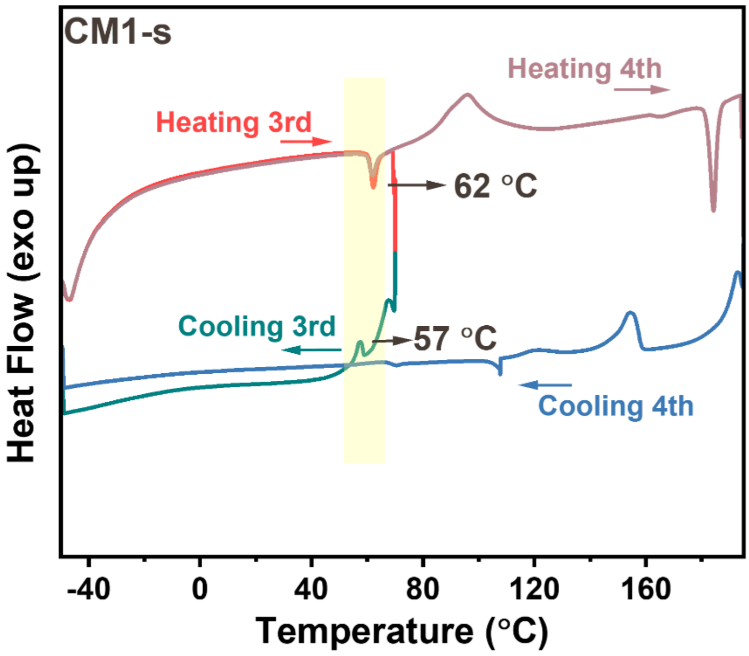

Supplementary Fig. S16| DSC thermograph of CM1-*s*. Heating rate: 10 ºC min^{-1}. The first three heating-cooling cycles are within the temperature range between 70 ºC and -50 ºC. The curves are repeatable and overlapped with each other, showing an endothermic peak at 62 ºC and an exothermic peak at 57 ºC, indicating the reversible phase change between CM1-*s* and CM1*-*s*. The fourth heating-cooling cycle (temp. range: -50-195 ºC) displayed a similar endothermic peak at 62 ºC, followed by an exothermic peak centered at ~ 95 ºC corresponding to the thermally activated topochemical polymerization. This result confirms that the endothermic peak at 62 ºC should be assigned to the reversible phase change due to the monomer crystal rearrangement. The additional endothermic peak at 182 ºC and exothermic peak at 157 ºC corresponds to an unidentified reversible phase changes of CP1-*s*.

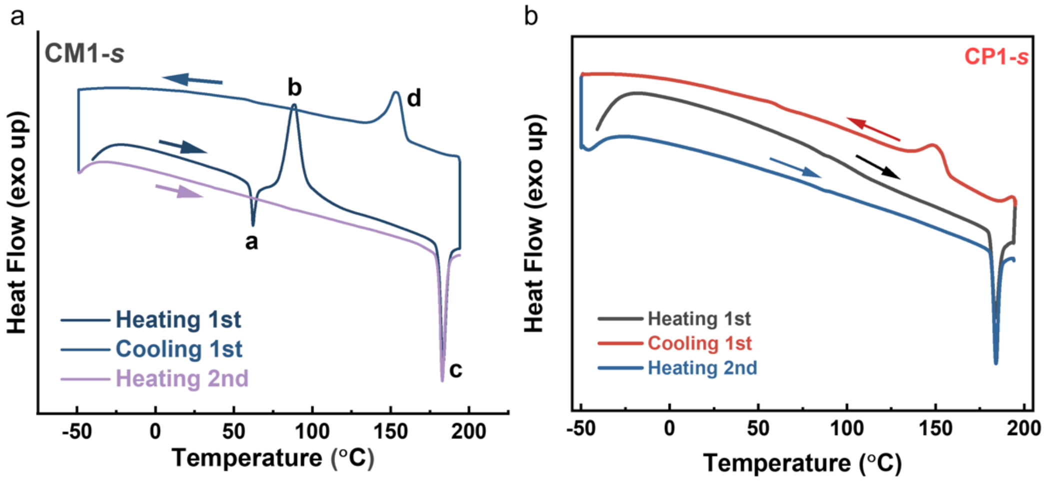

Supplementary Fig. S17| DSC curves of (a) CM1-*s* monomer and (b) CP1-*s* polymer. The sharp endothermic peaks observed in the first and second cycles could be assigned to the polymer phase change behavior. After polymerization, CP1-*s* exhibited a repeatable endothermic peak at ~180 °C, along with a broad exothermic peak at ~157 °C upon cooling. These phenomena correspond to a reversible thermally induced phase change in the polymer crystals.

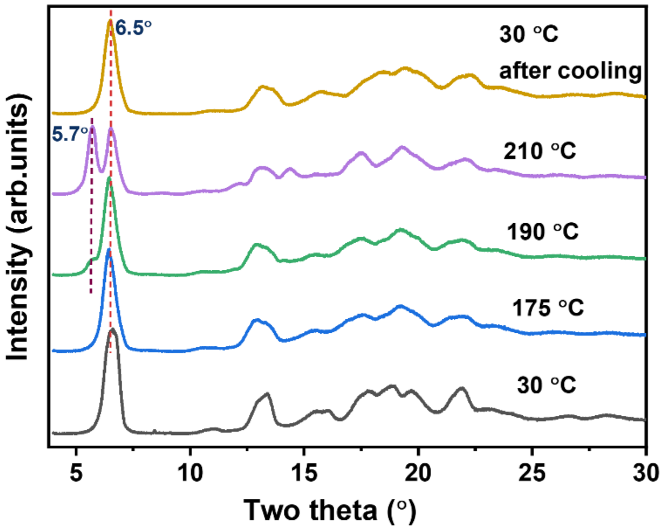

Supplementary Fig. S18| Variable-temperature PXRD of CP1-*s*, showing a reversible phase change at high temperatures.

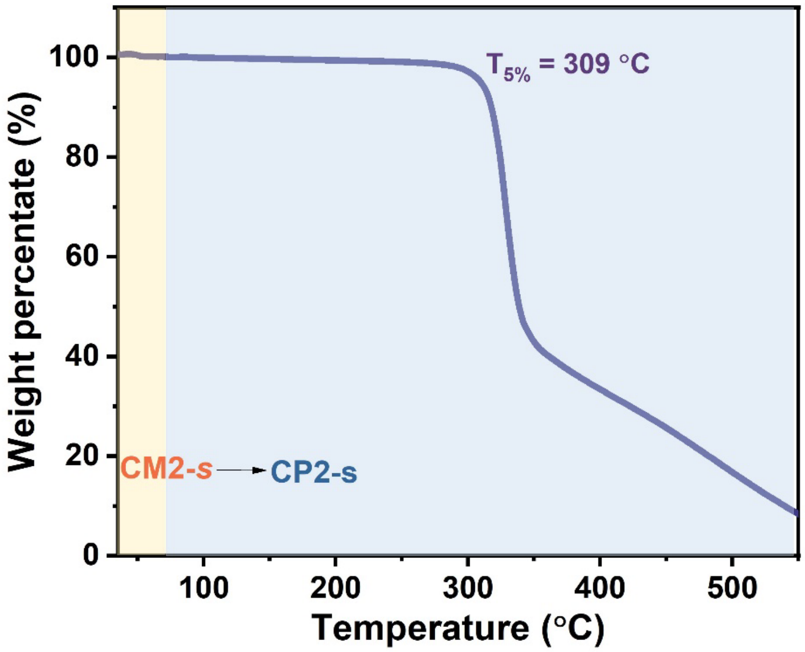

Supplementary Fig. S19| TGA characterization of CM2-*s* under nitrogen atmosphere. There is almost no weight loss before 309 ºC ($T_{5\%}$), indicating the high thermal stability of CP2-*s*.

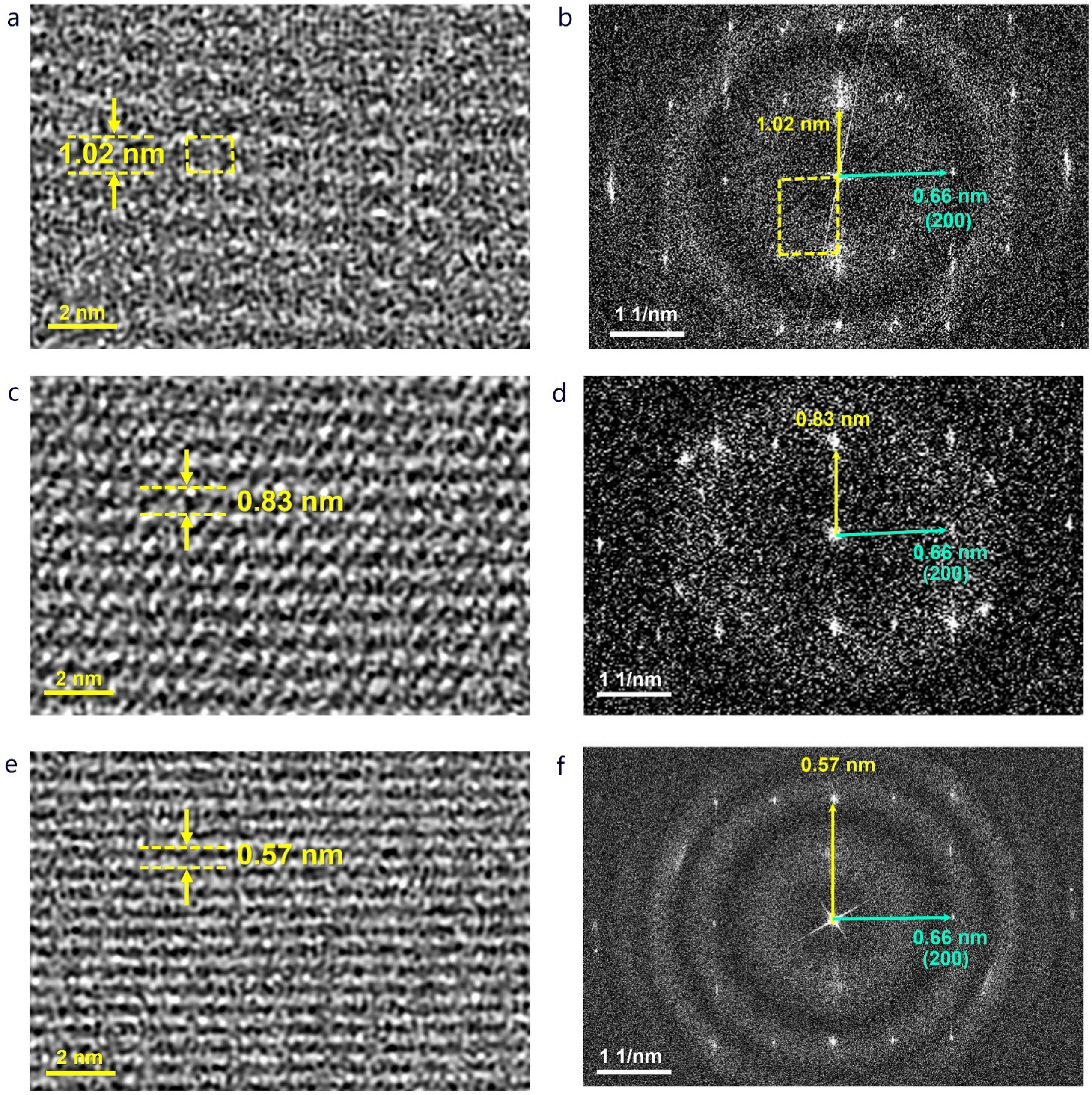

Supplementary Fig. S20| High-resolution cryo-EM images and corresponding FFT analysis of polycrystalline CP1-*s* at different orientations. The inter-chain separations are 1.02 nm (a, b), 0.83 nm (c, d) and 0.57 nm (e, f). The main-chain periodicity remains 1.32 nm despite different orientations.

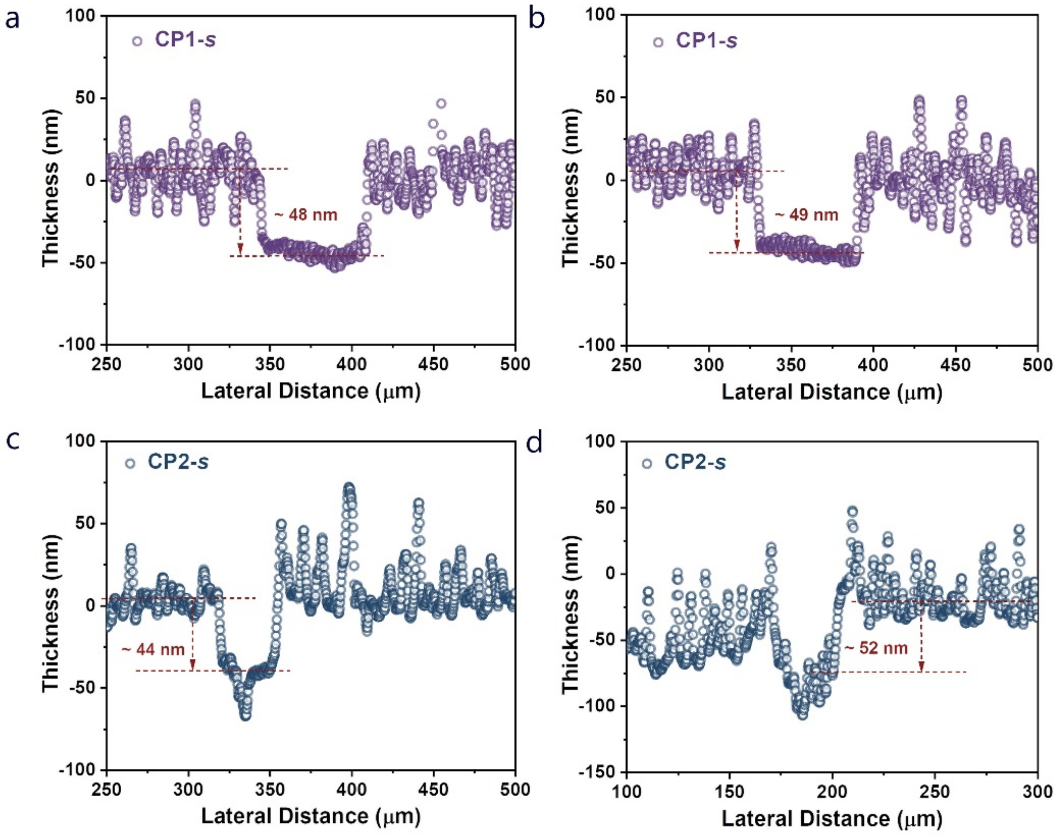

Supplementary Fig. S21| Thicknesses of CP1-*s* and CP2-*s* thin films measured by profilometry.

a-b, CP1-*s*. **c-d,** CP2-*s*. Average thickness are calculated to be ~ 50 nm for both CP1-*s* and CP2-*s*.

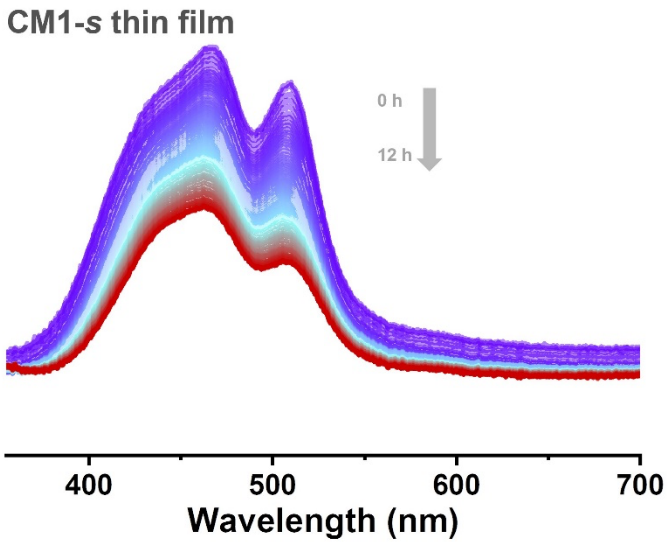

Supplementary Fig. S22| UV-vis absorption profiles of CM1-*s* thin film on a quartz substrate taken at regular intervals within ~12h. The measurements were carried out in the dark, so the exposure to light was limited to instrument excitation scans during data acquisition. Clear topochemical transformation could be observed with the gradual absorbance decay in the range of 400-600 nm, due to the loss of the feature of the AQM chromophore.

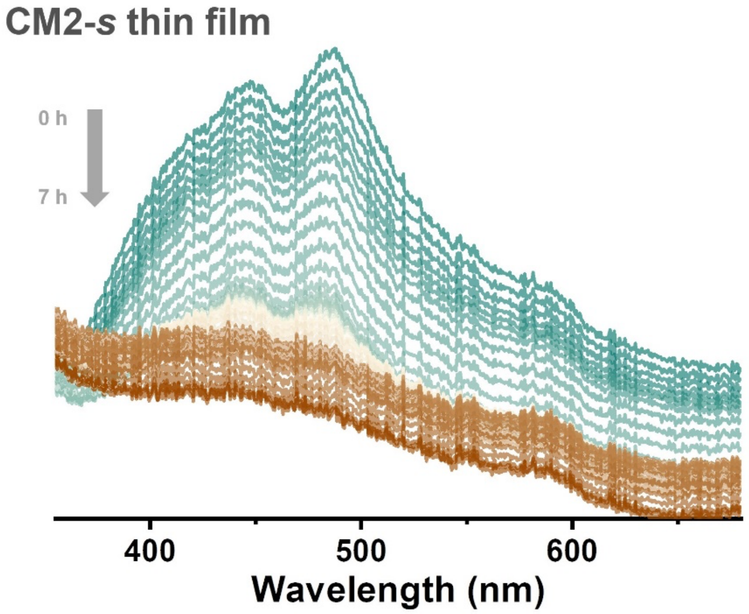

Supplementary Fig. S23| UV-vis absorption profiles of CM2-*s* thin film on a quartz substrate taken at regular intervals within ~ 7 h. The measurements were carried out in the dark, so the exposure to light was limited to instrument excitation scans during data acquisition. Clear topochemical transformation could be observed with the gradual absorbance decay in the range of 400-600 nm, due to the loss of the feature of the AQM chromophore.

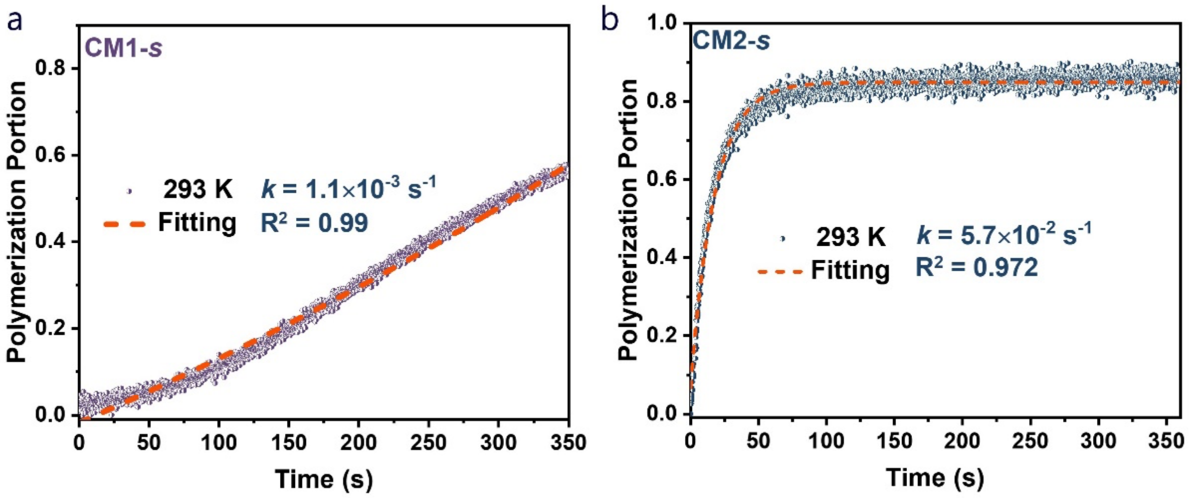

Supplementary Fig. S24| Polymerization versus irradiation time curves for (a) CM1-*s* and (b) CM2-*s* thin films at 293 K. The dashed line is the fitting curve based on the pseudo-first order kinetics. Corresponding reaction rates are calculated to be 1.1×10^{-3} and 5.7×10^{-2} s^{-1} for CM1-*s* and CM2-*s*, respectively.

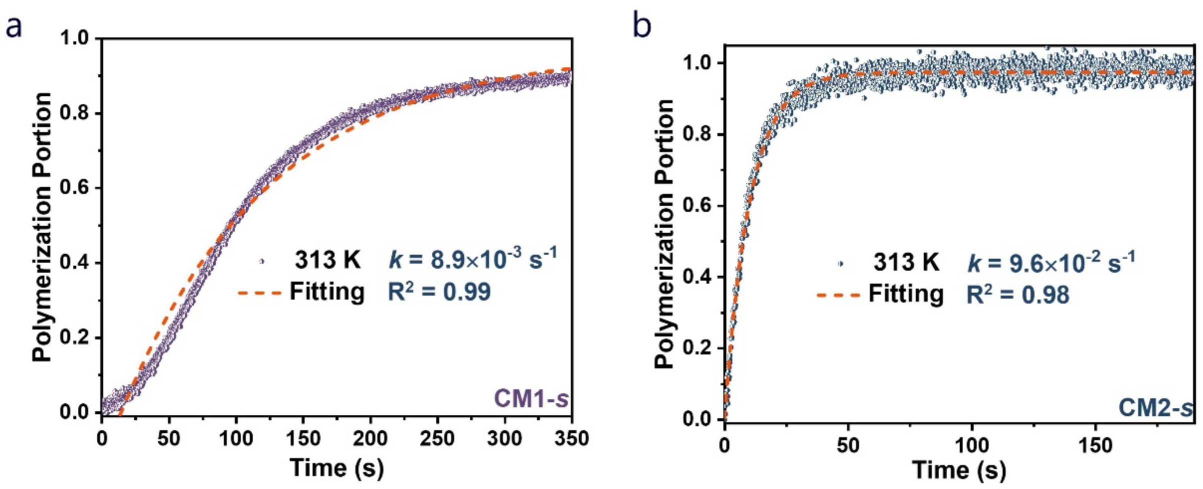

Supplementary Fig. S25| Polymerization versus irradiation time curves for (a) CM1-*s* and (b) CM2-*s* thin films at 313 K. The dashed line is the fitting curve based on the pseudo-first order kinetics. Corresponding reaction rates are calculated to be 8.9×10^{-3} and 9.6×10^{-2} s^{-1} for CM1-*s* and CM2-*s*, respectively.

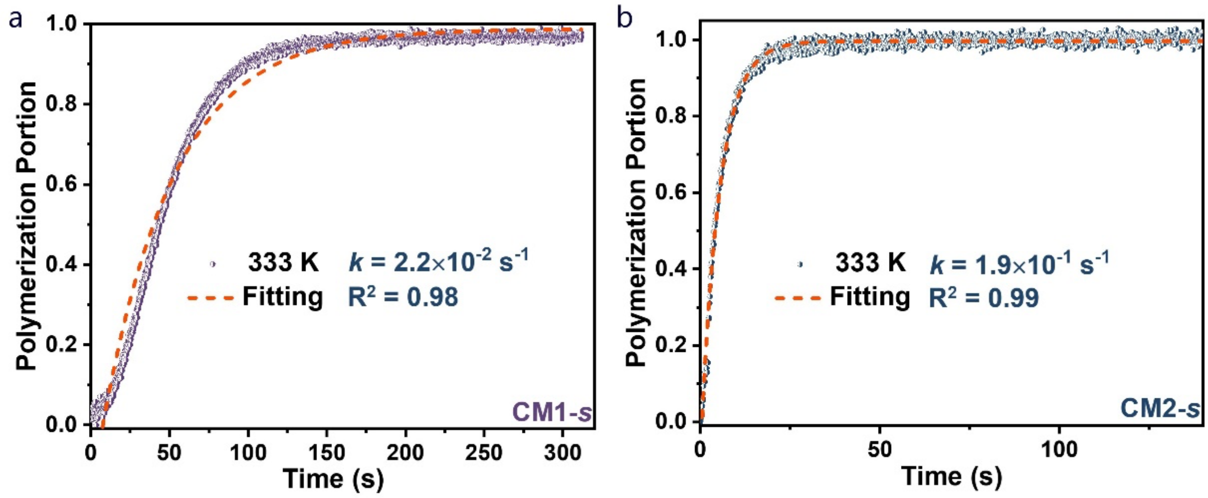

Supplementary Fig. S26| Polymerization versus irradiation time curves for (a) CM1-*s* and (b) CM2-*s* thin films at 333 K. The dashed line is the fitting curve based on the pseudo-first order kinetics. Corresponding reaction rates are calculated to be 2.2×10^{-2} and 1.9×10^{-1} s^{-1} for CM1-*s* and CM2-*s*, respectively.

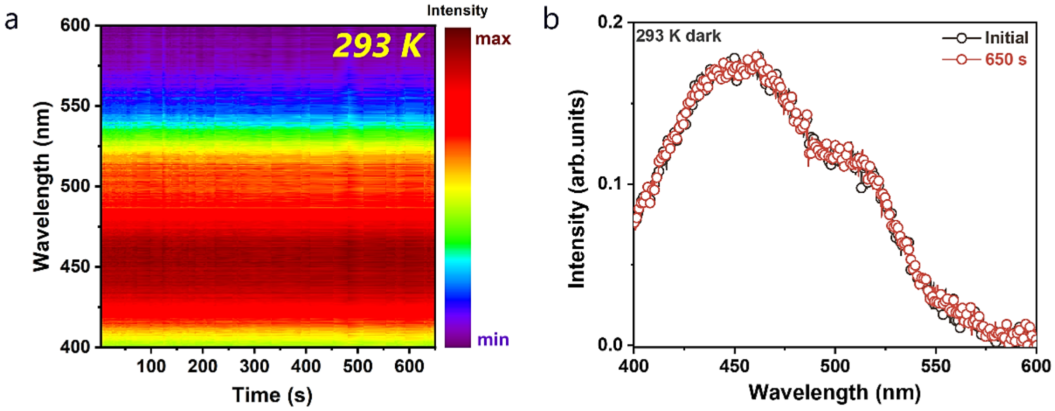

Supplementary Fig. S27| Absorption profiles of CM1-*s* at 293 K in the dark over a period of 650 s. a, False-color 2D plot of time-lapsed UV-vis absorption curves for CM1-*s* at 293 K in the dark. **b,** the corresponding 1D UV-vis absorption spectra of the film at 0 s and 650 s. There is no change in the absorption features during the test, confirming that there is negligible background reaction induced by the acquisition scan.

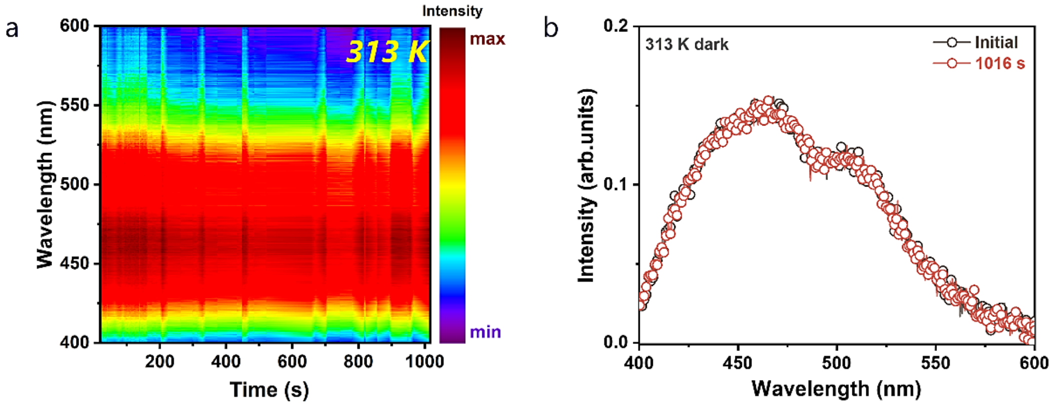

Supplementary Fig. S28| Absorption profiles of CM1-*s* at 313 K in the dark over a period of 1016 s. a, False-color 2D plot of time-lapsed UV-vis absorption curves for CM1-*s* at 313 K in the dark. The intermittent artifact was originated from the recurrent switching of light sources during the data acquisition across different wavelengths. **b,** Corresponding 1D UV-vis absorption spectra at 0 s and 1016 s. There is no absorbance change during the test, confirming that there is negligible background reaction induced by the acquisition scan at 313 K.

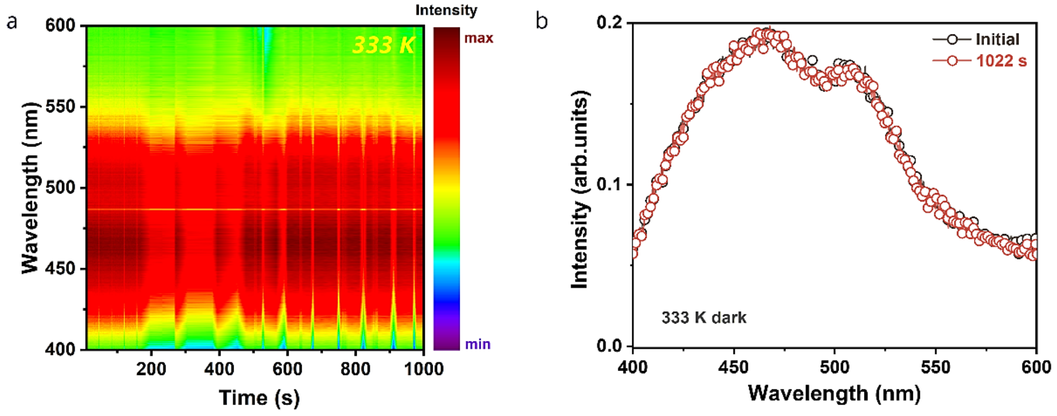

Supplementary Fig. S29| Absorption profiles of CM1-*s* at 333 K in the dark over a period of 1022 s. a, False-color 2D plot of time-lapsed UV-vis absorption curves for CM1-*s* at 333 K in the dark. The intermittent artifact was originated from the recurrent switching of light sources during the data acquisition across different wavelengths. **b,** Corresponding 1D UV-vis absorption spectra at 0 s and 1022 s. There is no absorbance change during the test, confirming that there is negligible background reaction induced by the acquisition scan at 333 K.

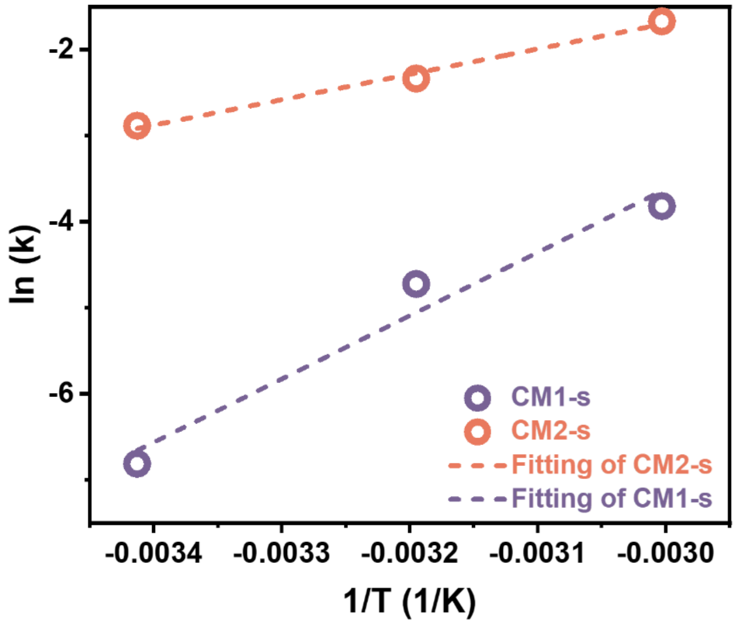

Supplementary Fig. S30| Activation energies for the TCP reaction of CM1-*s* and CM2-*s* obtained from fitting the Arrhenius equation. Corresponding activation energy were calculated to be 14.6 and 5.9 kcal mol^{-1} for CM1-*s* and CM2-*s*, respectively.

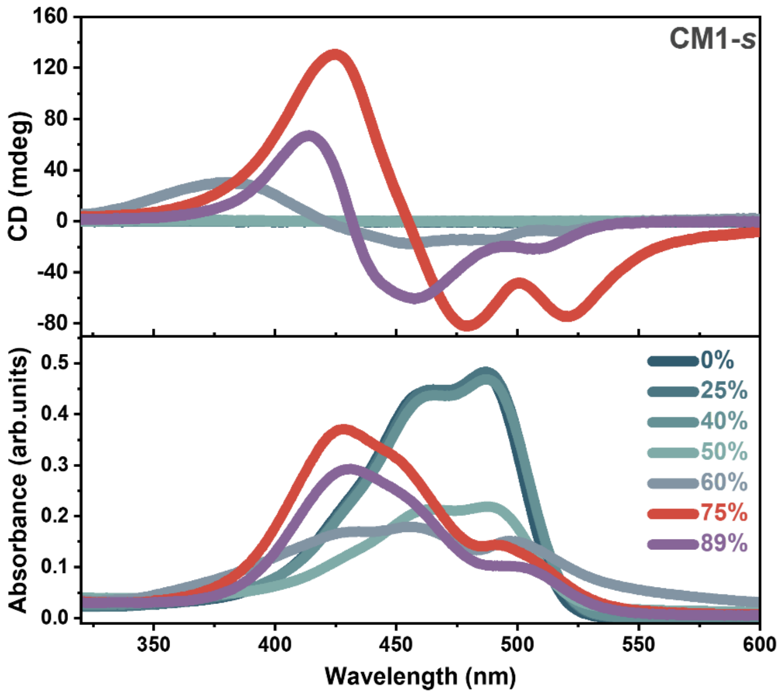

Supplementary Fig. S31| UV-vis and CD spectra of CM1-*s* in THF/H_2O at different solvent compositions. The concentration was 14 μM while the percentage of H_2O was varied from 0% to 89%.

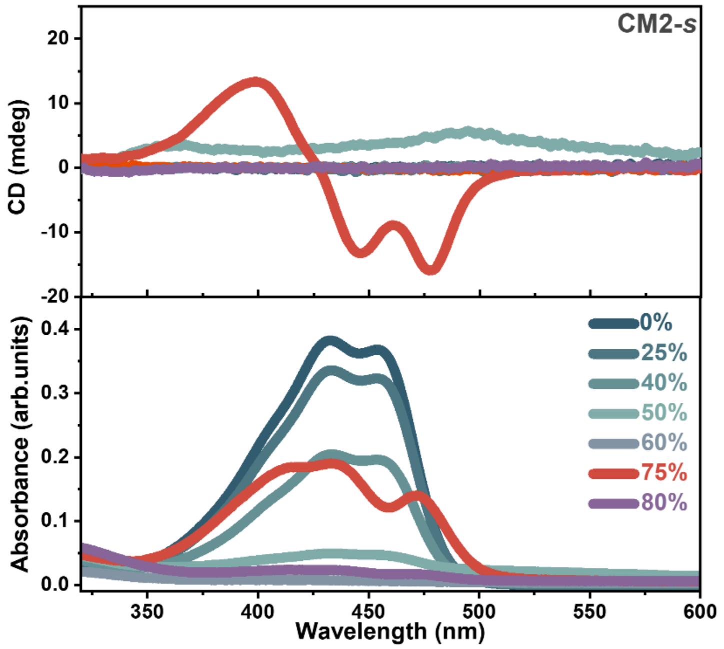

Supplementary Fig. S32| UV-vis and CD spectra of CM2-*s* in THF/H_2O at different solvent compositions. The concentration was 14 μM while the percentage of H_2O was varied from 0% to 80%.

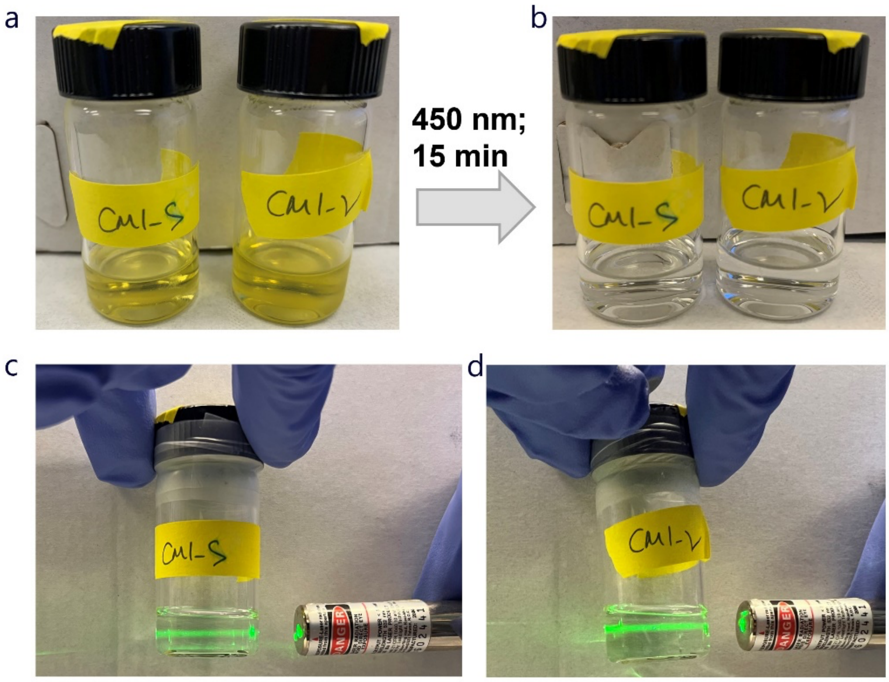

Supplementary Fig. S33| Photograph of antisolvent-driven self-assembly of CM1-*s*/*r* and subsequent TCP in aggregates. a, Photograph of antisolvent-driven self-assembly of CM1-*s*/*r*. **b,** Photograph of CM1-*s*/*r* after TCP in aggregates, generating colloidal CP1-*s*/*r* solutions. Obvious color change from yellow to colorless was observed after photopolymerization. Photographs showing Tyndall effect of the resulting colloidal solutions of (**c**) CP1-*s* and (**d**) CP1-*r*.

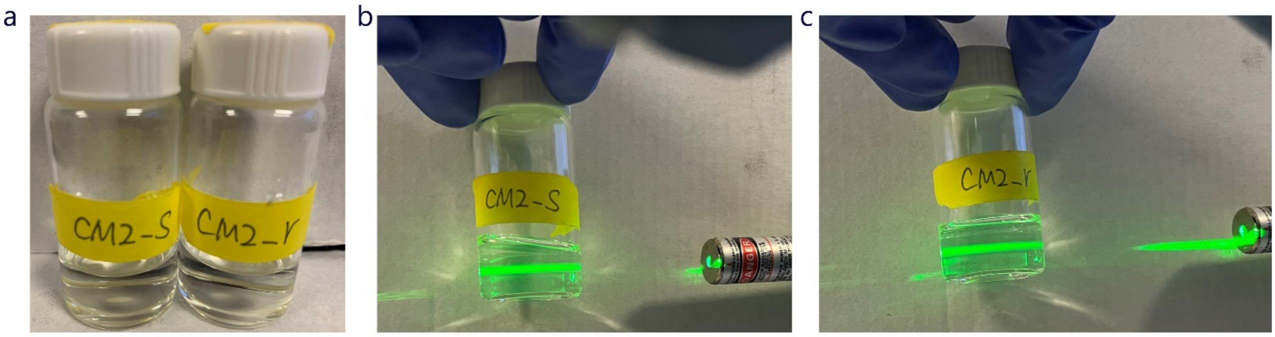

Supplementary Fig. S34| Photograph of CM2-*s*/*r* after TCP in aggregates. a, Photograph of CM2-*s*/*r* after TCP in aggregates, generating colloidal CP2-*s*/*r* solutions. Photographs showing Tyndall effect of the resulting colloidal solutions of (**b**) CP2-*s* and (**c**) CP2-*r*.

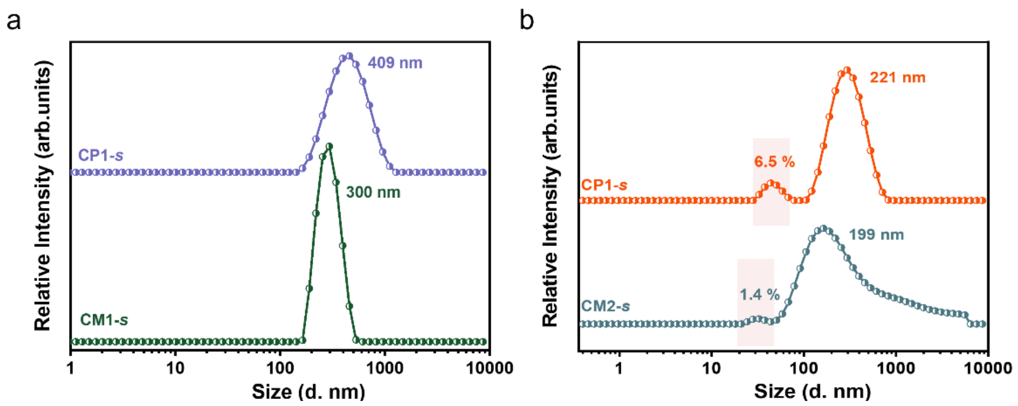

Supplementary Fig. S35| Size distribution determined by dynamic light scattering. a, CM1-*s* and CP1-*s*; **b,** CM2-*s* and CP2-*s*. The average hydrodynamic diameters (D_h) of CM1-s and CM2-s were determined to be ~ 300 nm and 199 nm, respectively. Following irradiation with 450 nm light, the D_h of the resulting CP1-s and CP2-s solutions increased to 409 nm and 221 nm, respectively. Due to the rapid polymerization kinetics of CM2-*s*, the broad peak of CM2-*s*, along with a small side peak centered at D_h = 33 nm (1.4%), was attributed to likely spontaneous TCP occurring during the DLS scanning process.

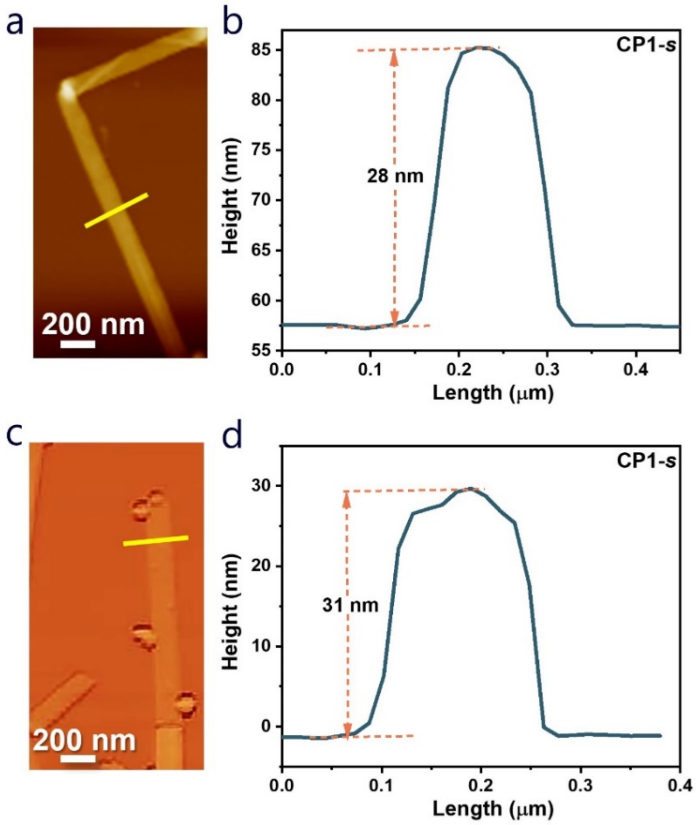

Supplementary Fig. S36| AFM images of (a) and (c) CP1-*s* nanofibers, and (b) and (d) corresponding height profiles. The height of thinnest CP1-*s* nanofibers is ~ 28 nm.

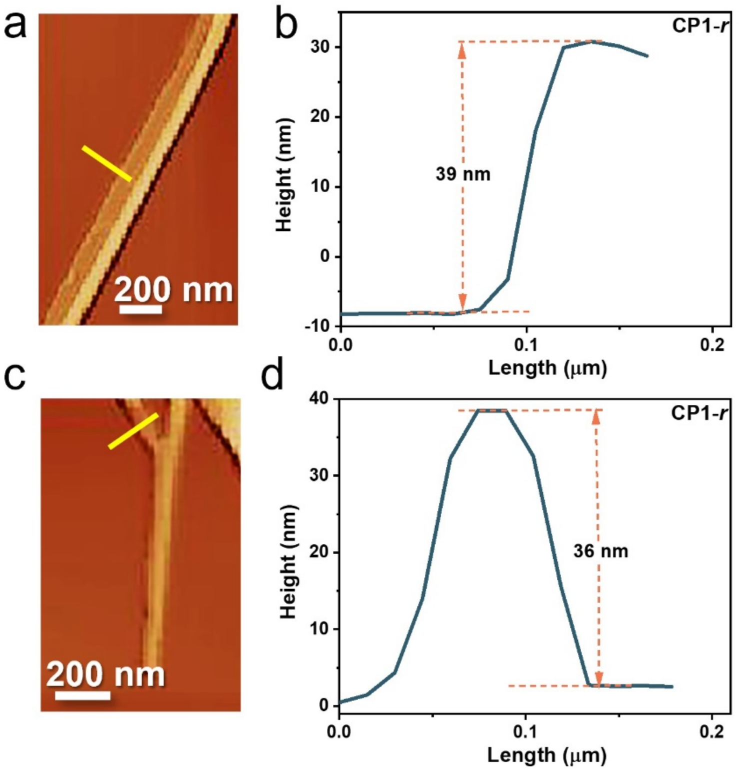

Supplementary Fig. S37| AFM images of (a) and (c) CP1-*r* nanofibers, and (b) and (d) corresponding height profiles. The height of thinnest CP1-*r* nanofibers is ~ 36 nm.

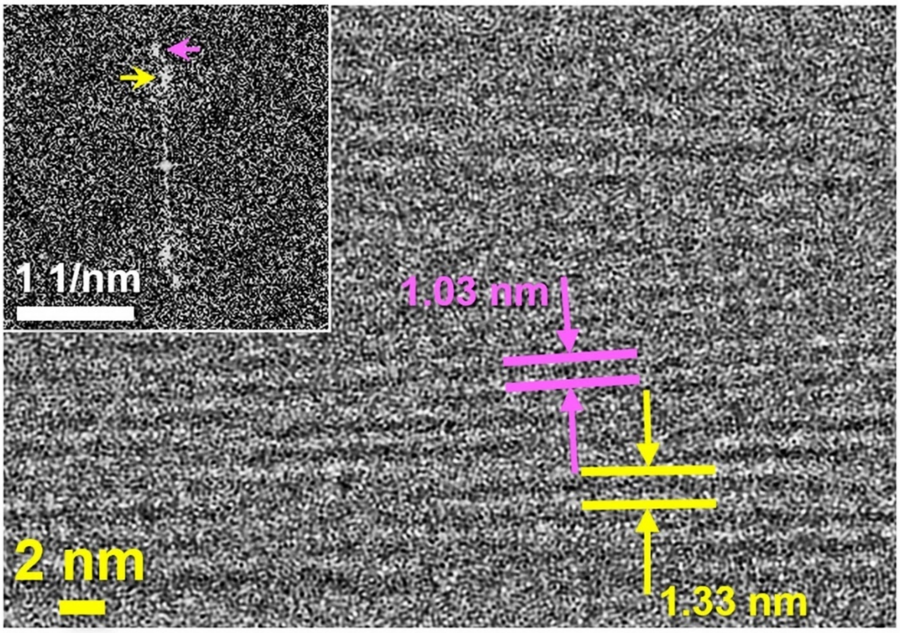

Supplementary Fig. S38| High-resolution cryo-EM image and corresponding FFT of a CP1-s nanofiber showing inter-chain separations of 1.33 nm and 1.03 nm.

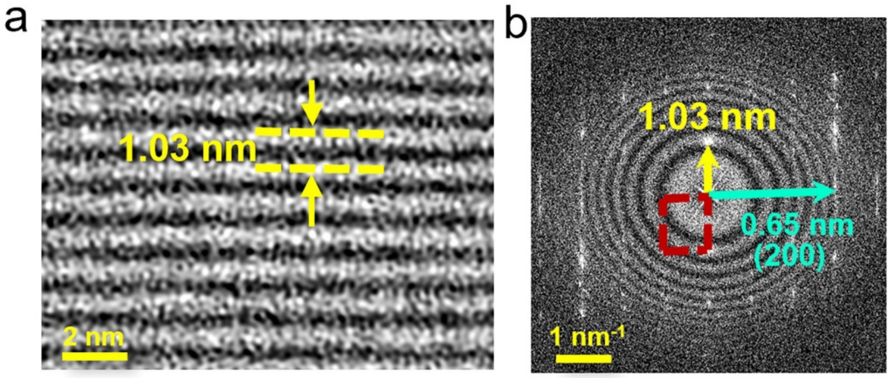

Supplementary Fig. S39| High-resolution cryo-EM image of a CP1-s nanofiber showing the packed polymer chain structures with distance of 1.03 nm (a) and corresponding FFT image (b).

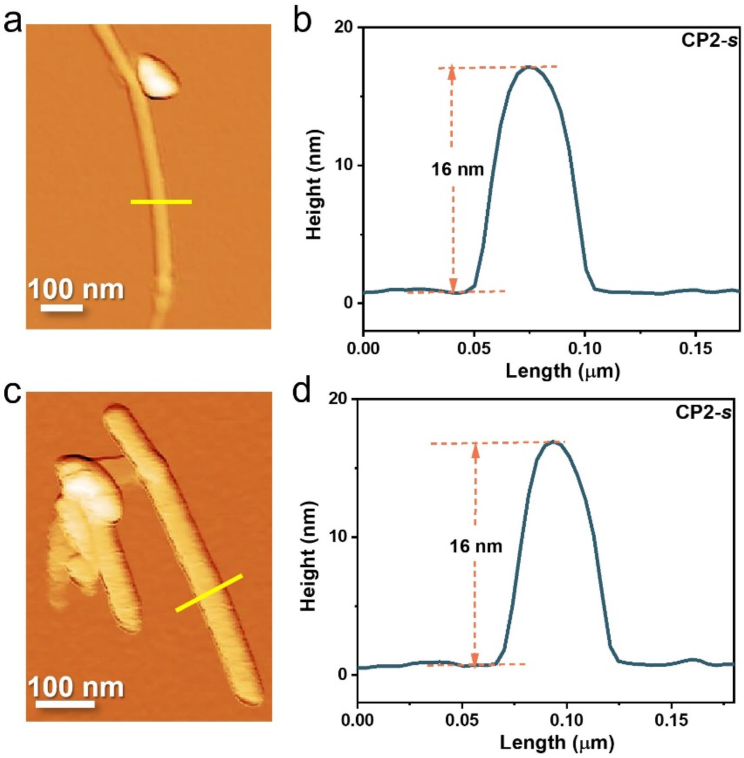

Supplementary Fig. S40| AFM images of (a) and (c) CP2-*s* nanofibers, and (b) and (d) corresponding height profiles. The height of thinnest CP2-*r* nanofibers is ~ 16 nm.

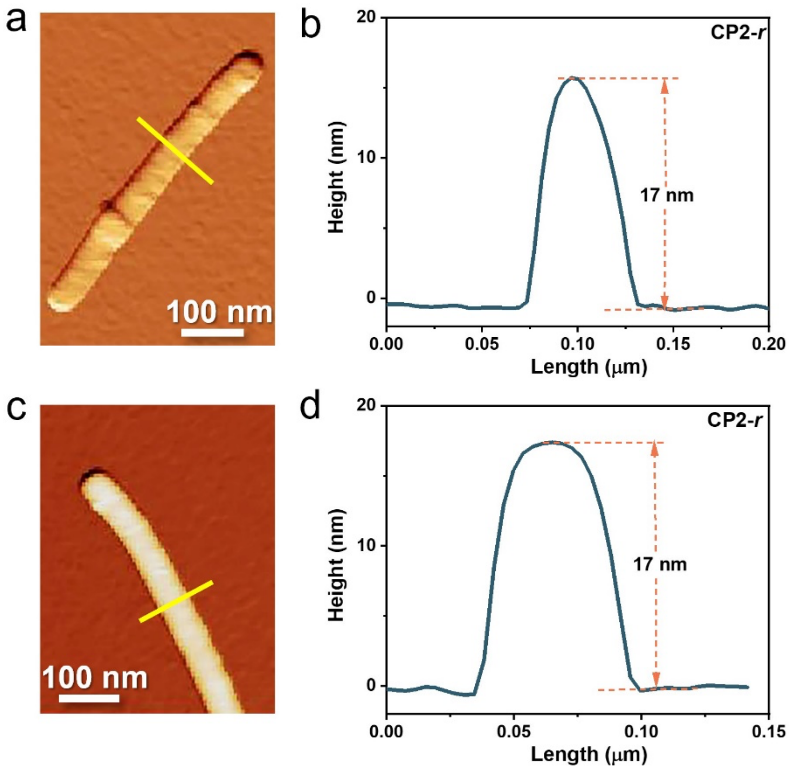

Supplementary Fig. S41| AFM images of (a) and (c) CP2-*r* nanofibers, and (b) and (d) corresponding height profiles. The height of thinnest CP2-*r* nanofibers is ~ 17 nm.

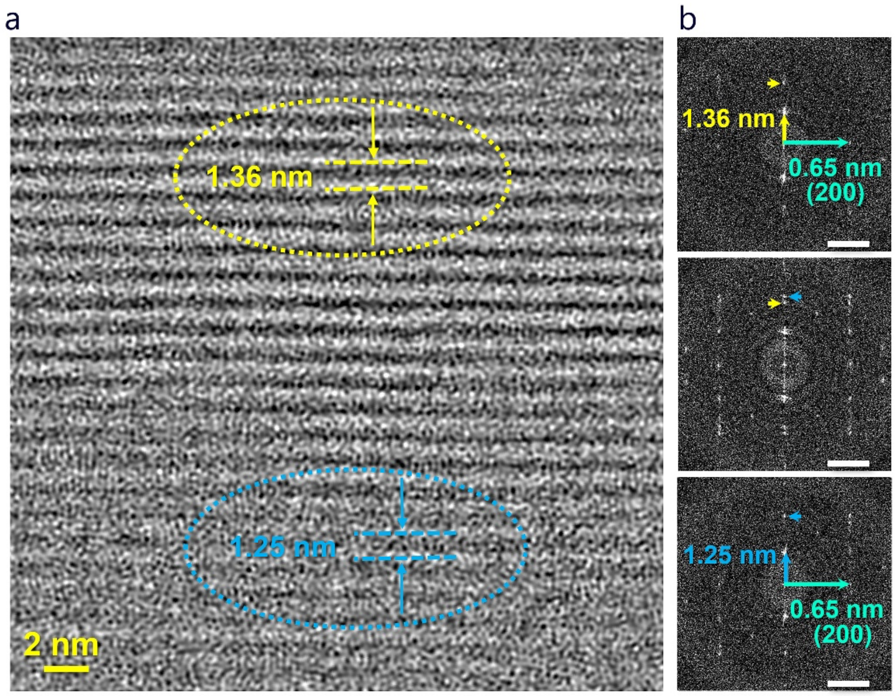

Supplementary Fig. S42| High-resolution cryo-EM images of (a) CP2-*s* nanofibers and (b) corresponding FFT from top area (left), full area (center) and bottom area (right). The results indicated two inter-chain separations of 1.36 nm and 1.25 nm associated with the same main-chain periodicity of 1.30 nm.

3. Supplementary Tables

Table S1 Crystallographic parameters for CM1-*s* and CM1-*r*

	**CM1-*s***	**CM1-*r***
**Empirical formula**	$C_{30}H_{32}F_6N_2O_8S_2$	$C_{30}H_{32}F_6N_2O_8S_2$
**Formula weight**	726.69	726.69
**Crystal system**	monoclinic	monoclinic
**Space group**	$P\ 2_1$	$P\ 2_1$
**a/ Å, b/ Å, c/ Å**	9.8273(5), 32.4681(17), 10.8474(6)	9.8277(7) 32.460(2) 10.8497(7)
**α/°, β/°, γ/°**	90.000, 111.831(3), 90.000	90.000, 111.753(2), 90.000
**Volume / Å^3**	3212.9(3)	3214.6(4)
**Z**	4	4
**ρ_{calc} / mg mm^{-3}**	1.502	1.433
**μ / mm^{-1}**	2.294	0.253
**F (000)**	1504	1504
**2θ range for data collection**	5.444 to 133.152°	4.042 to 50.698°
**Radiation**	CuKα (λ = 1.54178)	MoKα (λ = 0.71073)
**Index ranges**	$-11 \leq h \leq 11$, $-38 \leq k \leq 38$, $-12 \leq l \leq 12$	$-11 \leq h \leq 11$, $-39 \leq k \leq 39$, $-12 \leq l \leq 12$
**Reflections collected**	65905	59418
**Independent reflections**	11288 [R_{int} = 0.0419] [R_{sigma} = 0.0349]	11654 [R_{int} = 0.0737] [R_{sigma} = 0.0588]
**Data/restraints/parameters**	11288/1/873	11654/1/874
**Goodness-of-fit on F^2**	1.042	1.037
**Final R indexes [I>2σ (I)]**	R_1=0.0297, wR_2= 0.0817	R_1=0.0710, wR_2=0.1739
**Final R indexes [all data]**	R_1=0.0322, wR_2=0.0834	R_1= 0.1285, wR_2= 0.2235
**CCDC deposition number**	2359881	2359880

Table S2 Crystallographic parameters for CM2-*s* and CM2-*r*

	**CM2-*s***	**CM2-*r***
**Empirical formula**	$C_{40}H_{52}F_6N_2O_{10}S_2$	$C_{40}H_{52}F_6N_2O_{10}S_2$
**Formula weight**	898.95	898.95
**Crystal system**	triclinic	monoclinic
**Space group**	P 1	P 1
**a/ Å, b/ Å, c/ Å**	5.5230(5), 13.9267(13), 14.2513(14)	5.5237(6), 13.9156(14), 14.2774(15)
**α/°, β/°, γ/°**	83.544(4), 85.305(4), 89.138(4)	83.533(4), 85.283(4), 89.119(4)
**Volume / $Å^3$**	1085.54(18)	1086.72
**Z**	1	1
**ρ_{calc} / mg mm^{-3}**	1.375	1.374
**μ / mm^{-1}**	0.218	0.217
**F (000)**	472	472
**2θ range for data collection**	2.958 to 56.48°	3.02 to 59.00°
**Radiation**	Synchrotron (λ = 0.7288)	Synchrotron (λ = 0.7288)
**Index ranges**	$-7 \leq h \leq 7$, $-18 \leq k \leq 18$, $-18 \leq l \leq 18$	$-7 \leq h \leq 7$, $-18 \leq k \leq 18$, $-19 \leq l \leq 19$
**Reflections collected**	29326	30252
**Independent reflections**	9873 [R_{int} = 0.0865] [R_{sigma} = 0.1092]	11024 [R_{int} = 0.0707] [R_{sigma} = 0.0863]
**Data/restraints/parameters**	9873/3/549	11024/3/549
**Goodness-of-fit on F^2**	1.048	1.054
**Final R indexes [I>2σ (I)]**	R_1=0.0585, wR_2= 0.1453	R_1=0.0537, wR_2=0.1359
**Final R indexes [all data]**	R_1=0.1064, wR_2=0.1662	R_1=0.0815, wR_2= 0.1502
**CCDC deposition number**	2359878	2359879

Table S3 Crystallographic parameters for CM1*-*s* and CP1-*s*

	**CM1*-*s***	**CP1-*s***
**Empirical formula**	$C_{30}H_{32}F_6N_2O_8S_2$	$C_{30}H_{32}F_6N_2O_8S_2$
**Formula weight**	726.69	726.69
**Crystal system**	monoclinic	monoclinic
**Space group**	$P\ 2_1$	$P\ 2_1$
**a/ Å, b/ Å, c/ Å**	5.4369(7), 32.422(4), 9.2953(13)	6.38(3), 29.68(11), 8.89(3)
**α/°, β/°, γ/°**	90.000, 100.973(4), 90.000	90.000, 97.51(5), 90.000
**Volume / Å^3**	1608.6	1669.0
**Z**	2	2
**ρ_{calc} / mg mm^{-3}**	1.500	1.446
**μ / mm^{-1}**	0.253	0.259
**F (000)**	752	752
**2θ range for data collection**	4.464 to 58.266°	2.814 to 38.526°
**Radiation**	MoKα (λ = 0.71073)	Synchrotron (λ = 0.7288)
**Index ranges**	-7≤ h ≤7, -44≤ k ≤44, -12 ≤ l ≤12	-5≤ h ≤5, -26≤ k ≤26, -8≤ l ≤7
**Reflections collected**	63905	27861
**Independent reflections**	8551 [R_{int} = 0.0409] [R_{sigma} = 0.0251]	2571 [R_{int} = 0.2529] [R_{sigma} = 0.1115]
**Data/restraints/parameters**	8551/61/489	2571/319/434
**Goodness-of-fit on F^2**	1.073	1.161
**Final R indexes [I>2σ (I)]**	R_1= 0.0659, wR_2= 0.1748	R_1=0.1596, wR_2=0.3598
**Final R indexes [all data]**	R_1= 0.0725, wR_2= 0.1820	R_1=0.2017, wR_2= 0.4066
**CCDC deposition number**	2359882	2359883

Table S4 Crystallographic parameter change during the SCSC polymerization

	**CM1-*s* to CM1*-*s***	**CM1*-*s* to CP1-*s***	**CM1-*s* to CP1-*s***
**a**	-44.7%	17.3%	-35.1%
**b**	-0.2%	-8.5%	-8.6%
**c**	-14.3%	-4.4%	-18.1%
**α**	0	0	0
**β**	-9.7%	-3.4%	-12.8%
**γ**	0	0	0
**Volume**	-49.9%	3.8%	-48.1%

4. NMR spectra of CM1-s/r and CM2-s/r

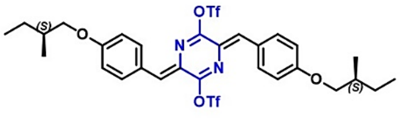

CM1-s

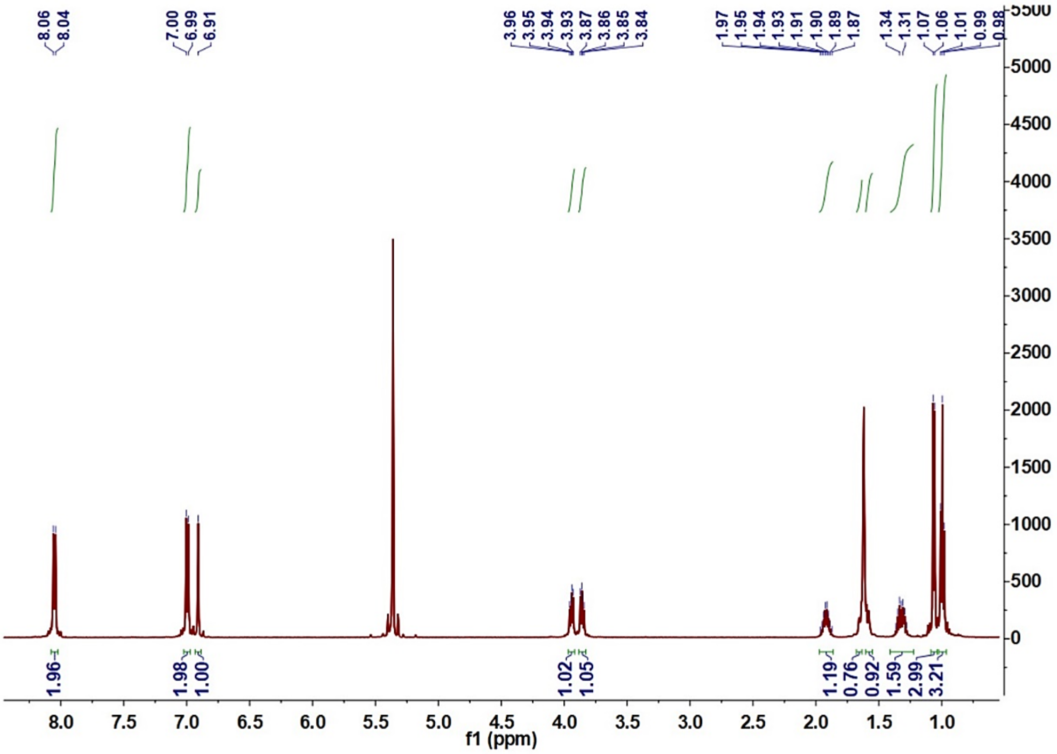

Supplementary Fig. S43| ^{1}H NMR Spectrum of CM1-*s* (CD_2Cl_2, 500 MHz, 298K).

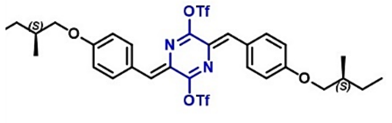

CM1-*s*

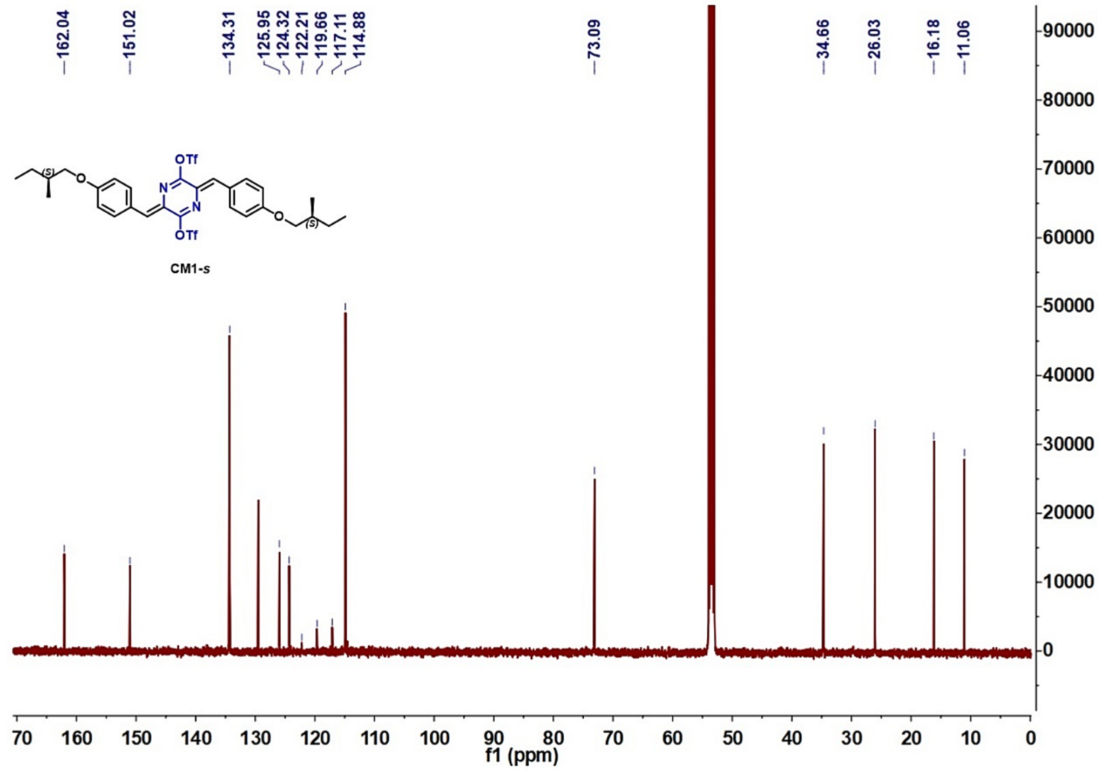

Supplementary Fig. S44| ^{13}C NMR Spectrum of CM1-*s* (CD_2Cl_2, 126 MHz, 298K).

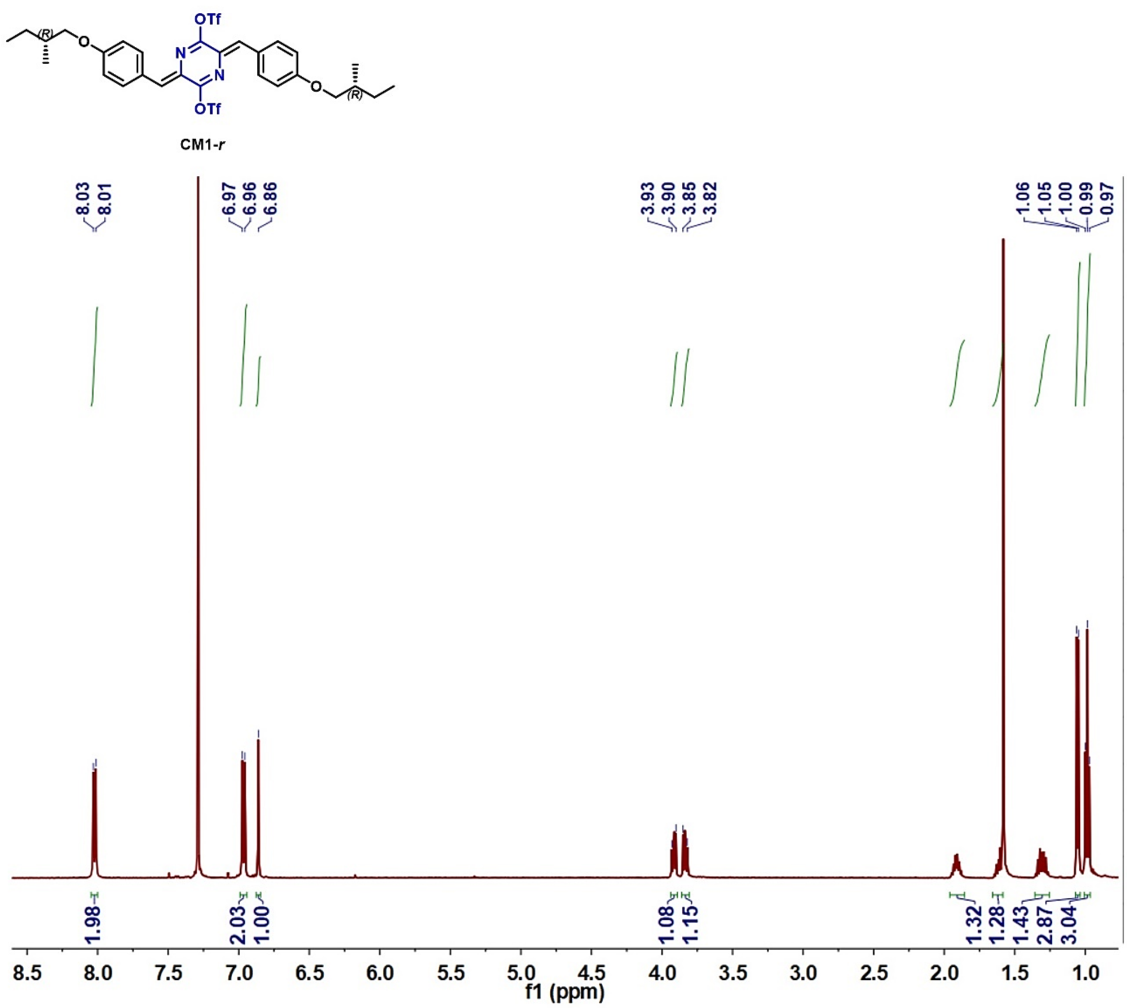

Supplementary Fig. S45| ^{1}H NMR Spectrum of CM1-*r* ($CDCl_3$, 500 MHz, 298K).

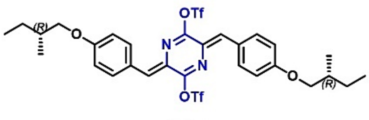

CM1-*r*

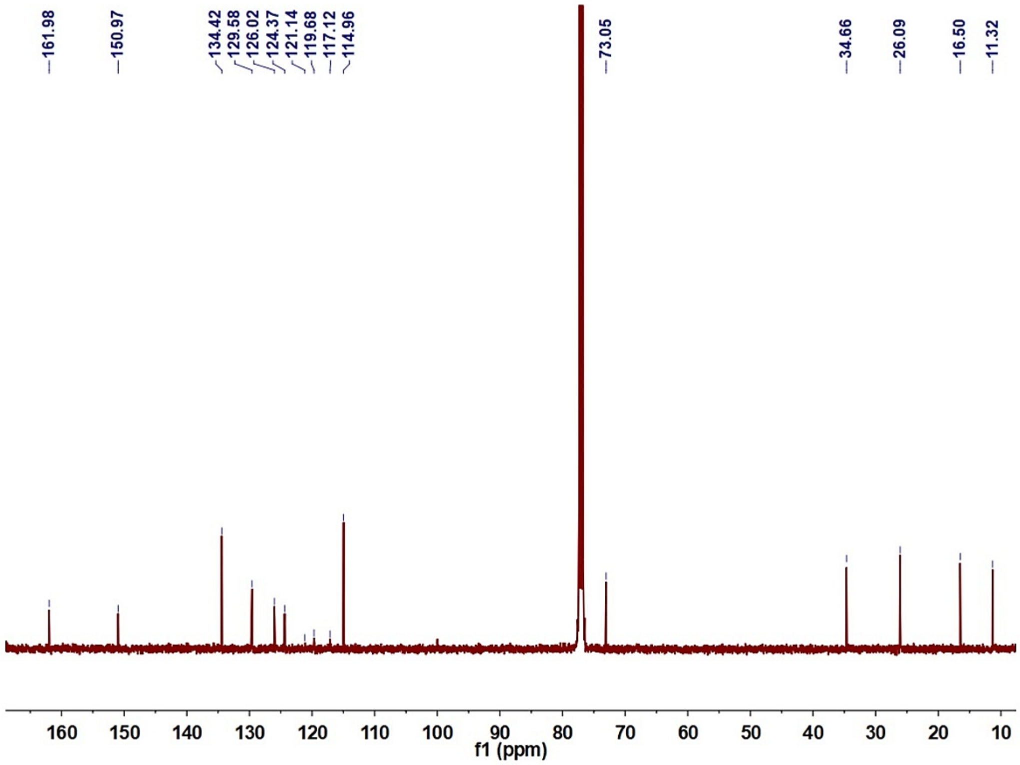

Supplementary Fig. S46| ^{13}C NMR Spectrum of CM1-*r* ($CDCl_3$, 126 MHz, 298K).

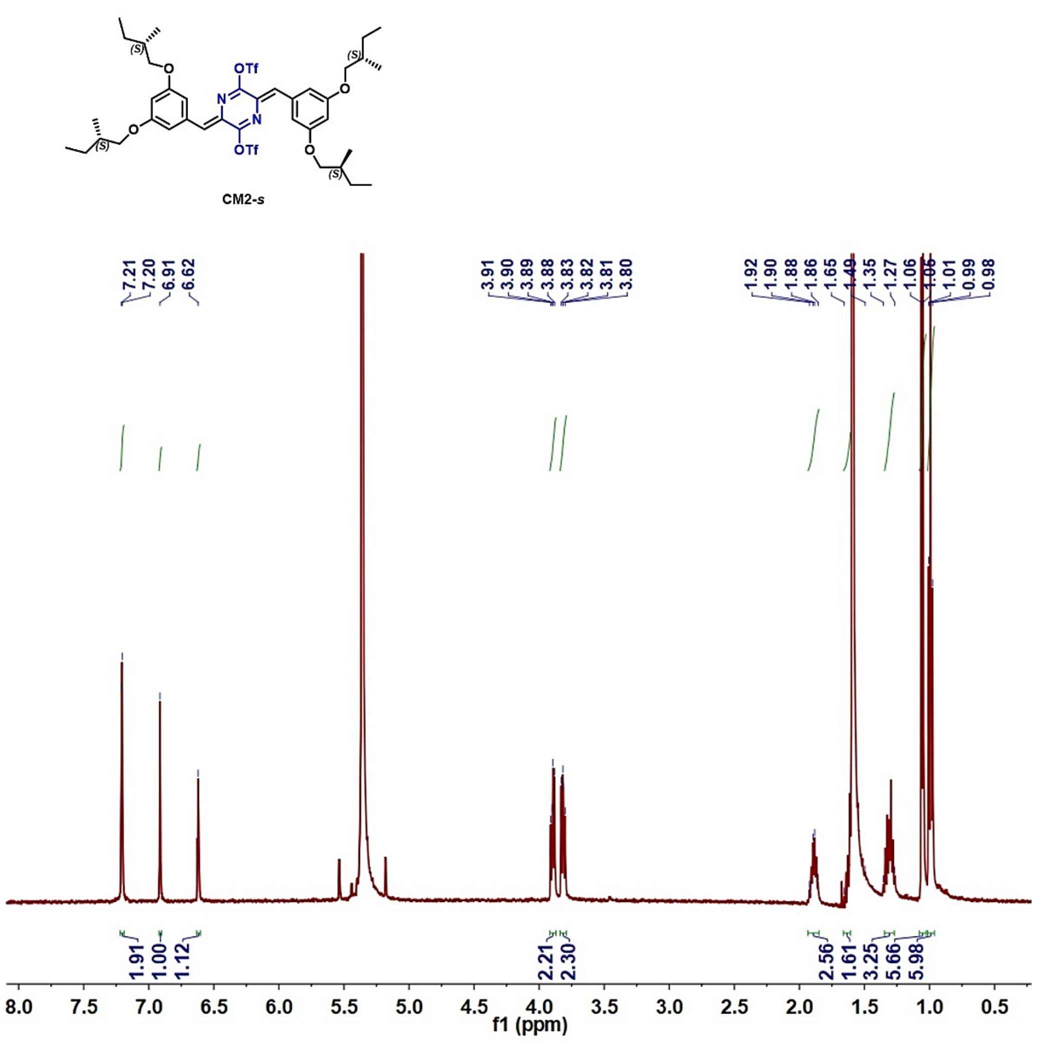

Supplementary Fig. S47| ^{1}H NMR Spectrum of CM2-*s* (CD_2Cl_2, 500 MHz, 298K).

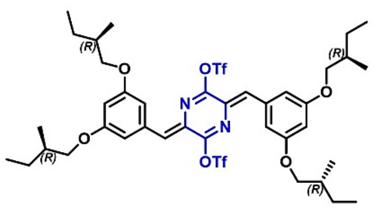

CM2-*r*

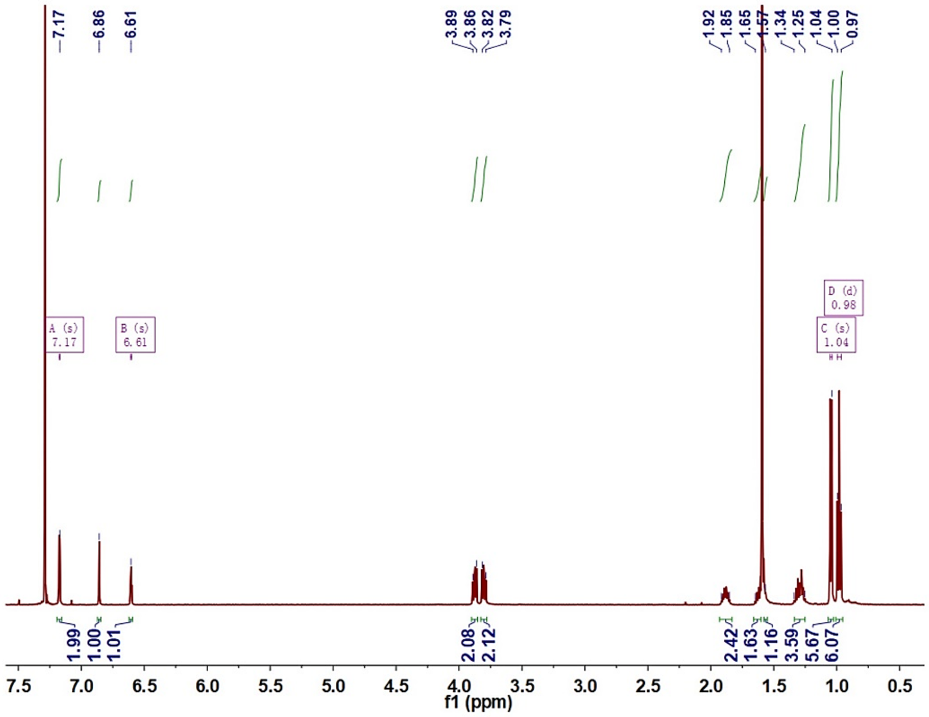

Supplementary Fig. S48| ^{1}H NMR Spectrum of CM2-*r* ($CDCl_3$, 500 MHz, 298K).

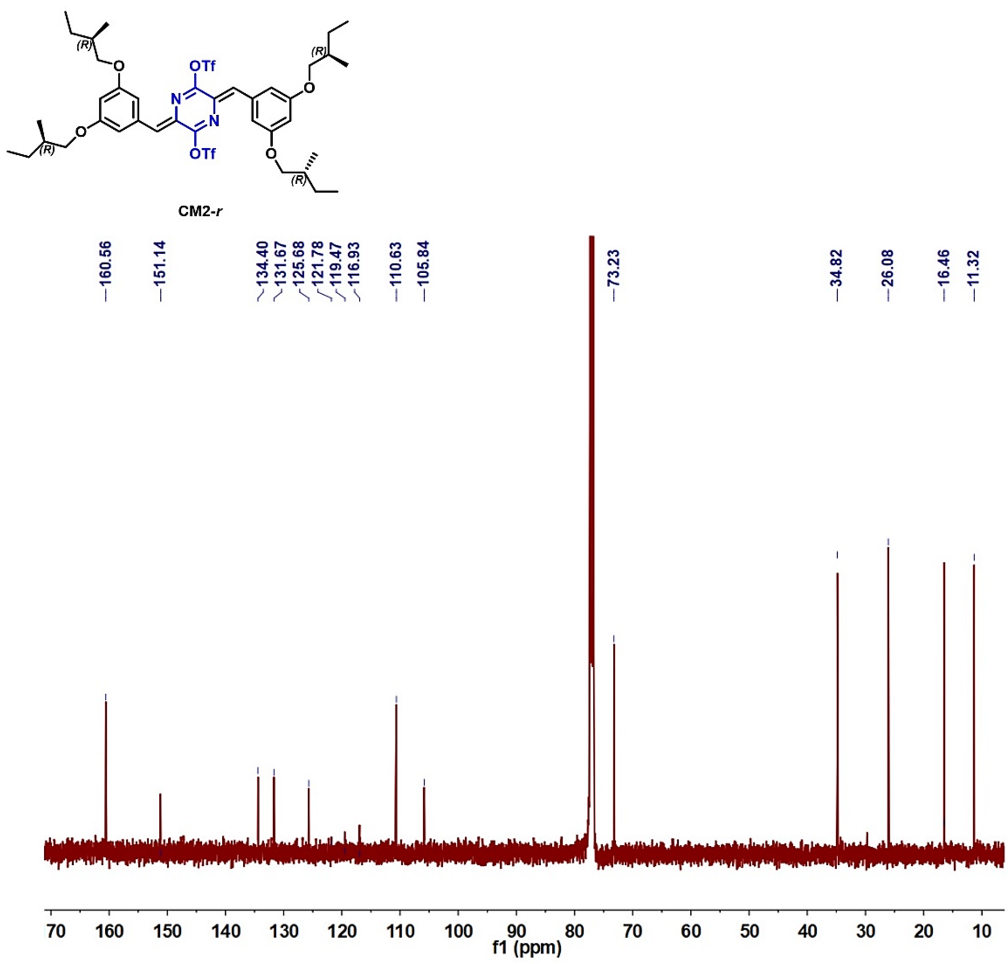

Supplementary Fig. S49| ^{13}C NMR Spectrum of CM2-*r* ($CDCl_3$, 126 MHz, 298K).

5. Reference

1. Anderson, C. L. *et al.* Solution-processable and functionalizable ultra-high molecular weight polymers via topochemical synthesis. *Nat. Commun.* **12**, 6818 (2021).
2. Becke, A. D. et al. Density-functional exchange-energy approximation with correct asymptotic behavior. *Phys. Rev.* A **38**, 3098 (1988).
3. Lee, C. T. et al. Development of the Colle-Salvetti correlation-energy formula into a functional of the electron density. *Phys. Rev.* B **37**, 785 (1988).
4. Grimme, S. et al. A consistent and accurate ab initio parametrization of density functional dispersion correction (DFT-D) for the 94 elements H-Pu. *J. Chem. Phys.* **132**, 154104 (2010).
5. Ditchfield, R. et al. Self-consistent molecular-orbital methods. IX. An extended gaussian-type basis for molecular-orbital studies of organic molecules. *J. Chem. Phys.* **54**, 724 (1971).
6. Hehre, W. J. et al. Self-consistent molecular orbital methods. XII. Further extensions of gaussian-type basis sets for use in molecular orbital studies of organic molecules. *J. Chem. Phys.* **56**, 2257 (1972).
7. Hariharan, P. et al. The influence of polarization functions on molecular orbital hydrogenation energies. *Theor. Chem. Acc.* **28**, 213 (1973).
8. Francl, M. M. et al. Self-consistent molecular orbital methods. XXIII. A polarization-type basis set for second-row elements. *J. Chem. Phys.* **77**, 3654 (1982).
9. Frisch, M. J. et al. Gaussian 16, Revision A.03, Gaussian, Inc., Wallingford CT, 2016.
10. Sheldrick, G. M. Phase annealing in SHELX-90: direct methods for larger structures. *Acta Crystallogr. A* **46**, 467-473 (1990).
