# Supplementary material for: High-fidelity topochemical polymerization in single crystals, polycrystals, and solution aggregates
Source: Nat Commun. 2025 Apr 12;16:3498. doi: 10.1038/s41467-025-58822-2 (PMC11993752; doi:10.1038/s41467-025-58822-2)
Supplement: Supplementary file 3 — Description of Additional Supplementary Files [file 41467_2025_58822_MOESM3_ESM.pdf]

Description of Additional Supplementary Files

File Name: Supplementary Movie 1
Description: Photo-induced polymerization of a crystal of CM1-s
Under continuous exposure to ambient light, a single crystal of CM1-s underwent photo-induced polymerization that eventually led to crystal disintegration.
